# Hydrophilic Random Cationic Copolymers as Polyplex-Formation Vectors for DNA

**DOI:** 10.3390/ma15072650

**Published:** 2022-04-04

**Authors:** Varvara Chrysostomou, Hector Katifelis, Maria Gazouli, Konstantinos Dimas, Costas Demetzos, Stergios Pispas

**Affiliations:** 1 Section of Pharmaceutical Technology, Department of Pharmacy, School of Health Sciences, National and Kapodistrian University of Athens, Panepistimioupolis Zografou, 15771 Athens, Greece; vchrysost@pharm.uoa.gr; 2 Theoretical and Physical Chemistry Institute, National Hellenic Research Foundation, 48 Vassileos Constantinou Avenue, 11635 Athens, Greece; 3 Laboratory of Biology, Medical School, National and Kapodistrian University of Athens, Michalakopoulou 176, 11527 Athens, Greece; e-ktor@hotmail.com (H.K.); mgazouli@med.uoa.gr (M.G.); 4 Second Department of Radiology, Medical School, National and Kapodistrian University of Athens, 11527 Athens, Greece; 5 Department of Pharmacology, Faculty of Medicine, School of Health Sciences, University of Thessaly, 41500 Larissa, Greece; ksdimas@yahoo.com

**Keywords:** non-viral vectors, gene delivery, nucleic acids, DNA, random copolymers, polyplexes, cationic polymers, RAFT polymerization, in vitro cytotoxicity

## Abstract

Research on the improvement and fabrication of polymeric systems as non-viral gene delivery carriers is required for their implementation in gene therapy. Random copolymers have not been extensively utilized for these purposes. In this regard, double hydrophilic poly[(2-(dimethylamino) ethyl methacrylate)-co-(oligo(ethylene glycol) methyl ether methacrylate] [P(DMAEMA-co-OEGMA)] random copolymers were synthesized via reversible addition-fragmentation chain transfer (RAFT) polymerization. The copolymers were further modified by quaternization of DMAEMA tertiary amine, producing the cationic P(QDMAEMA-co-OEGMA) derivatives. Fluorescence and ultraviolet–visible (UV–vis) spectroscopy revealed the efficient interaction of copolymers aggregates with linear DNAs of different lengths, forming polyplexes, with the quaternized copolymer aggregates exhibiting stronger binding affinity. Light scattering techniques evidenced the formation of polyplexes whose size, molar mass, and surface charge strongly depend on the N/P ratio (nitrogen (N) of the amine group of DMAEMA/QDMAEMA over phosphate (P) groups of DNA), DNA length, and length of the OEGMA chain. Polyplexes presented colloidal stability under physiological ionic strength as shown by dynamic light scattering. In vitro cytotoxicity of the empty nanocarriers was evaluated on HEK293 as a control cell line. P(DMAEMA-co-OEGMA) copolymer aggregates were further assessed for their biocompatibility on 4T1, MDA-MB-231, MCF-7, and T47D breast cancer cell lines presenting high cell viability rates.

## 1. Introduction

The progress of molecular biology in the elucidation of the molecular structure of nucleic acids and the identification of their genetic profiles in biological functions, which are associated with several diseases, brought gene therapy to the forefront of modern medicine [1]. Nowadays, gene therapy is considered the most promising and revolutionary strategy for the treatment of a broad range of genetic-based diseases, even applicable to individualized medicine [2].

The successful implementation of gene therapy treatment options requires the appropriate design of gene delivery systems. By far, viral vectors have been extensively utilized for the delivery of genetic material due to their high therapeutic efficiency [1,3]. However, their safety issues regarding immunogenic responses [4] have motivated the development of non-viral gene delivery systems based on innovative nanomaterials.

In the research of these delivery systems, the potential benefits of polymeric carriers have attracted scientific interest for their further development and improvement as non-viral genetic material transfer agents, especially due to their better safety profile compared with viral vectors. Moreover, their unlimited payload capacity and controllable chemical diversity [5] permit the delivery of different macromolecular cargos (pDNA, siRNA, mRNA, and proteins) according to the requirements of each biomolecule for efficient intracellular delivery to target sites [6].

In particular, synthetic or natural cationic polymers [7,8] containing primary, secondary, and/or tertiary amine groups can electrostatically bind and condense nucleic acids in the form of nanoscaled polyplexes [1,6,9]. The polyplex formation is an entropy-driven process based on ionic interaction between the cationic amino groups and the negatively charged phosphate groups of polyanionic nucleic acids [6,10]. Polyplexes are able to protect the genetic material from enzymatic degradation and facilitate cellular uptake and intracellular release [11,12]. Furthermore, the complexation of negatively charged nucleic acids with a small excess of positively charged cationic polymers improves nucleic acid compaction and increases the positive surface charge [9]. Polyplexes with positive surface charge are more efficient in cell interaction through the electrostatic binding to the negatively charged cell membrane [1,5] and therefore promote enhanced intracellular uptake [6]. Hence, the ratio (N/P) of the positively charged polymer amine groups (N) to negatively charged nucleic acid phosphate groups (P), along with the surface charge, the size, and colloidal stability in biological fluids of the polyplexes are determinants of efficient transfection [8,9,12].

The stability of polyplexes in blood and other biological fluids is an issue of great importance. Their interaction with electrolytes, serum proteins, and polysaccharides can lead to the partial or total dissociation of the polyplexes before performing the required delivery function [1,9,13]. Moreover, depending on the ionic strength of biological media, the positively charged polyplexes tend to self-aggregate into larger structures which may result in limited in vivo applicability due to biocompatibility risks [14]. The major bottleneck of polyplexes is their inherent cytotoxicity due to the cationic character of the polymers utilized, which makes their clinical utilization a remaining challenge [6,11,12,15,16]. Moreover, the excess of positive charges provides high transfection efficiency but also enhances cytotoxicity effects [17]. Therefore, optimizing the N/P ratio, by achieving a delicate balance between high transfection efficiency, colloidal stability, and low toxicity profile [12,16], is significant for polyplex biological fate.

A common and efficient strategy to overcome these hurdles involves the modification of cationic polymers with the addition of non-ionic hydrophilic functionalities of poly(ethylene glycol) (PEG) moieties. The PEGylation of polyplexes has been shown to ameliorate cytotoxicity effects during transfection, due to the shielding of their surface charge [16,18]. Moreover, PEG modifications provide polyplexes with colloidal stability, prevent aggregation and unwanted interactions with serum proteins, reduce opsonization, and prolong their systemic circulation time [17,19,20]. However, shielding of polyplexes with PEG leads to lower transfection efficiencies and therefore reduced cellular uptake [6,12,21]. Alternative shielding agents close to commonly used PEG are polymers of oligo(ethylene glycol) methacrylates (POEGMAs) [6,20]. POEGMAs are composed of a hydrophobic methacrylate main chain and grafted hydrophilic side chains of oligo(ethylene glycol) moieties. Variation of the number of ethylene glycol (EG) repeated units, and therefore the length of the side chains can impact on the properties of OEGMA-based polymers. For instance, OEGMAs with short side chains (less than nine repeated units of ethylene glycol) present thermoresponsive behavior [20,22], while OEGMAs with long side chains provide increased solubility and enhanced stealth and shielding properties [23,24].

Poly[2-(dimethylamino)ethyl methacrylate] (PDMAEMA) is a highly promising cationic polymer for its implementation as an efficient non-viral gene delivery vector. The tertiary amine group of PDMAEMA is partially protonated (pK_a_ ca. 7.4) under physiological conditions (pH 7.4 and ionic strength 150 mM) [16,25], allowing the electrostatic interaction with nucleic acids and the formation of polyplexes. Moreover, PDMAEMA, due to its pK_a_, presents a high buffering capacity facilitating the endosomal escape of polyplexes through the proton sponge effect [16,25,26]. PDMAEMA exhibits less cytotoxicity compared with other cationic polymers and enhanced transfection efficiency [25,26,27]. Furthermore, the controllable synthetic preparation with tunable molecular characteristics [25] and various macromolecular architectures using controlled radical polymerization methodologies [6,25,26], the easy functionalization of its tertiary amine for high-binding affinity to nucleic acids [27,28], and the stimuli-responsive behavior to pH and temperature [29,30,31] render PDAEMA even more attractive for its utilization as a gene transfection vector. However, the cationic nature of PDMAEMA bears cytotoxicity issues [12,26] and colloidal instability [25] which are the major limiting factors for in vivo application. The cytotoxicity of PDMAEMA is also associated with its molar mass [12]. More specifically, PDMAEMA with a molar mass larger than 112 kDa exhibits increased cytotoxicity but at the same time high transfection efficiency [26]. In this regard, approaches to overcome these obstacles include the copolymerization with non-ionic hydrophilic monomers and PEGylation of PDMAEMA [19,26]. Indeed, PEGylation efficiently improves the cytotoxicity of PDMAEMA but also reduces its transfection efficiency [19,26]. Therefore, further optimization in the design of PDMAEMA polymers, without sacrificing its transfection efficiency, are necessary to achieve effective nucleic acid delivery.

The advances in controlled radical polymerization (CRP) techniques including atom transfer polymerization (ATRP) and reversible addition-fragmentation chain transfer (RAFT) polymerization have contributed significantly to the design of new polymeric gene vectors, providing precise macromolecular synthesis of polymers with predictable molecular architecture and narrow molar mass distributions [32,33,34]. Amongst CRP processes, RAFT polymerization is the most frequently used in gene delivery, due to the exceptional versatility that is offered in the fabrication of well-defined polymers with predetermined molecular characteristics [35,36]. Moreover, its compatibility with a variety of monomers [32], the potential of producing stimuli-responsive polymers with pH-sensitivity [37], the incorporation of functional groups such as active agents and targeting moieties [32,38], and the facile synthetic preparation under aqueous conditions [35] without the use of toxic metal catalysts [33] render RAFT polymerization an attractive technique for advanced gene delivery polymer systems. Furthermore, RAFT provides the potential of designing polymeric gene vectors with different and controlled architectures including homopolymers, block copolymers, random or statistical copolymers, graft and star-shaped copolymers [32]. The facile preparation of block copolymers and especially random copolymers with controllable molecular characteristics permit the potential of scale-up production, which is a significant criterion in the design of a gene delivery system.

In this study, we aimed to design polyplexes as nucleic acid delivery vectors based on cationic PDMAEMA copolymers with the non-ionic, hydrophilic, and biocompatible OEGMA. We utilized the OEGMA oligomer alternatively to PEG, with average M_n_ = 950 g/mol and 19 ethylene glycol repeated units, in an effort to reduce the cytotoxicity effects of the cationic PDMAEMA. Furthermore, the long-chain length of OEGMA is expected to provide enhanced stealth properties, biocompatibility, shielding effect, and colloidal stability to the polyplexes. Hence, we synthesized poly[(2-(dimethylamino) ethyl methacrylate)-co-(oligo(ethylene glycol) methyl ether methacrylate] [P(DMAEMA-co-OEGMA)] double hydrophilic random copolymers of low molecular masses, by utilizing RAFT polymerization. Moreover, the tertiary amine groups of DMAEMA segments were converted to quaternary ammonium salts with a permanent cationic charge. The P(DMAEMA-co-OEGMA) and P(QDMAEMA-co-OEGMA) copolymers were characterized by several techniques in order to determine their molecular and physicochemical characteristics. Moreover, the in vitro cytotoxicity of the copolymer aggregates was examined in different types of cell lines. Furthermore, we focus on the investigation of the ability of the P(DMAEMA-co-OEGMA) and P(QDMAEMA-co-OEGMA) random copolymer aggregates to interact with model DNA of different lengths and of linear topology. The binding affinity of copolymer aggregates was examined by spectroscopy techniques. Nanosized polyplexes were formed through the electrostatic interaction of the random copolymer aggregates with the DNAs, in a wide range of N/P ratios (nitrogen (N) of amine groups of cationic polymer over phosphate (P) groups of DNA). The physicochemical characteristics of the formed polyplexes, including their size and surface charge, as well as the influence of ionic strength on their colloidal stability, were examined by light scattering techniques.

## 2. Materials and Methods

### 2.1. Materials and Reagents

For the synthesis of the random copolymers, the monomer 2-(dimethylamino ethyl methacrylate) (DMAEMA, 98%), the reactive oligomer oligo(ethylene glycol) methyl ether methacrylate (OEGMA) with average M_n_ = 950 g/mol and 19 ethylene glycol units, the hydroquinone monomethyl ether (MEHQ) and butylated hydroxytoluene (BHT) inhibitor removers, the chain transfer agent 4-cyano-4-[(dodecyl sulfanylthio carbonyl)sulfanyl]pentanoic acid (CPD), the radical initiator 2,2-azobis(isobutyronitrile) (AIBN), methyl iodide (CH_3_I) as the quaternization agent, benzene (99.7%), tetrahydrofuran (THF 99.9%), 1,4-dioxane (99.8%), and deuterium oxide (D_2_O) (99.9%) were obtained from Sigma-Aldrich (Athens, Greece) and used as received, except 1,4-dioxane, which was dried over molecular sieves before use. AIBN was purified by recrystallization from methanol. Dialysis tubing membranes (MEMBRA-CEL^®^) from regenerated cellulose of MWCO 3500 and a diameter of 22 mm were obtained by SERVA (Heidelberg, Germany).

For in vitro cytotoxicity studies, the human embryonic kidney HEK293 cells (CRL-1573™), which were used as normal cell line, and the rat breast cancer 4T1 cells (CRL-2539™) were purchased from ATCC (Manassas, VA, USA). The human breast cancer cell lines MDA-MB-231, MCF-7, and T47D were obtained from NCI (NCI, NIH, Frederick, MD, USA). The DMEM cell culture medium, glutamine, penicillin, and streptomycin were obtained from BioSera (Nuaille, France), while fetal bovine serum (FBS) was from Pan-Biotech (Aidenbach, Germany). MTS assay (CellTiter 96^®^ Aqueous One Solution, Madison, WI, USA) was utilized as a colorimetric method for determining the number of viable cells and was purchased from Promega (Madison, WI, USA).

For the interaction of the copolymer aggregates with nucleic acids, linear deoxyribonucleic acid (DNA) sodium salt from salmon testes with a length 2000 bp was obtained from Sigma-Aldrich (Athens, Greece) and linear DNA sodium salt from salmon sperm with a length of 113 bp was purchased from Acros Organics (Geel, Belgium). Ethidium bromide (EtBr) dye for DNA quenching fluorescent assay was received from Sigma-Aldrich (Athens, Greece). Sodium chloride (99.0%) was also received from Sigma-Aldrich (Athens, Greece) and used for the preparation of a purified NaCl solution of 0.01 M and 1 M. All the solutions were prepared using sterile water for injection (DEMO SA., Athens, Greece).

### 2.2. Synthesis of Copolymers and Their Quaternized Derivatives

Reversible addition-fragmentation chain transfer polymerization (RAFT) was employed for the synthesis of two P(DMAEMA-co-OEGMA) random copolymers, varying in molecular mass and composition. The following procedure for the synthesis of the copolymers is described in detail below. DMAEMA and OEGMA were purified by passing through columns packed with hydroquinone monomethyl ether (MEHQ) and butylated hydroxytoluene (BHT) inhibitor removers before the polymerization process. For the synthesis of P(DMAEMA-co-OEGMA)-1: in a round bottom flask (25 mL) equipped with a magnetic stirrer (Sigma-Aldrich, Athens, Greece), purified DMAEMA (0.5 g, 3.18 mmol), purified OEGMA (0.5 g, 0.53 mmol), 4-cyano-4-[(dodecyl sulfanylthio carbonyl)sulfanyl]pentanoic acid (CPD) (0.04 g, 0.01 mmol), 2,2-azobis(isobutyronitrile) (AIBN) (0.0016 g, 0.01 mmol) and 5 mL 1,4-dioxane (20 wt.% monomer solution) were added under stirring. The used CTA (CPD) to initiator (AIBN) ratio ([CTA]_0_/[I]_0_) was 10:1.

**Scheme 1:**
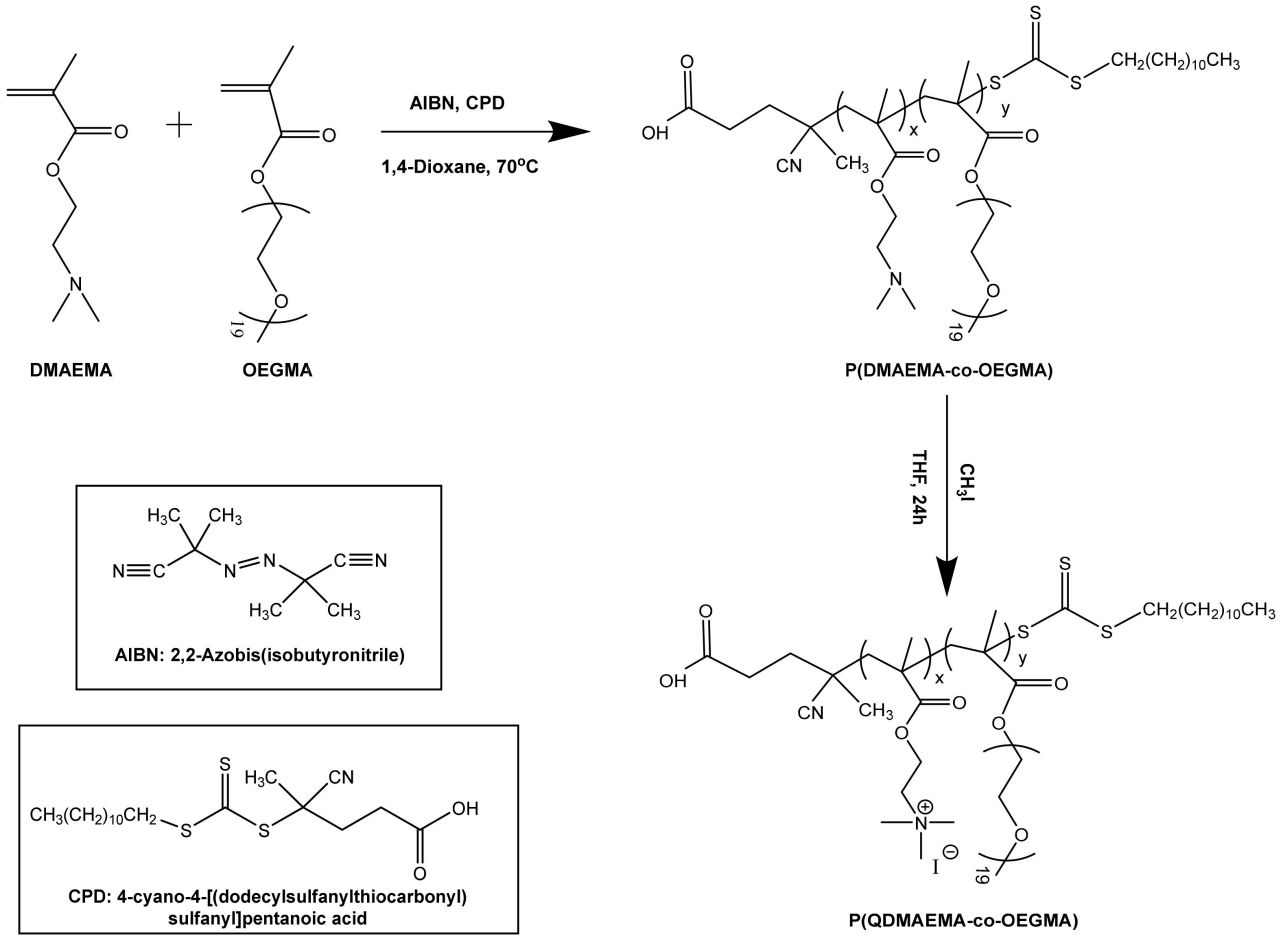
Synthetic route for the production of P(DMAEMA-co-OEGMA) copolymers and their quaternized P(QDMAEMA-co-OEGMA) derivatives.

The round bottom flask was sealed with a rubber septum. The reaction solution was degassed by high purity nitrogen gas bubbling for 20 min and then immersed in a preheated oil bath at 70 °C for 24 h. Afterward, the reaction was quenched by freezing at −20 °C for 30 min and exposing it to air. Subsequently, the reaction product was purified by dialysis against deionized H_2_O for 3 days in order to remove unreacted monomers and other impurities. The pure copolymer was isolated using a rotary evaporator and dried under a vacuum oven for 48 h at 25 °C. A similar synthetic procedure was followed for the synthesis of P(DMAEMA-co-OEGMA)-2. The followed synthetic polymerization route and the chemical structure of the copolymers is depicted in Scheme 1.

P(DMAEMA-co-OEGMA) copolymers were modified by the quaternization of their tertiary amine group to quaternized ammonium salt. The quaternization reaction was accomplished by the following process: 0.2 g of P(DMAEMA-co-OEGMA) copolymers were dissolved in 10 mL tetrahydrofuran (2% *w*/*v*) and were placed in a round bottom flask (25 mL). Afterward, an excess of CH_3_I (1.16 mmol for P(DMAEMA-co-OEGMA)-1 and 0.90 mmol for P(DMAEMA-co-OEGMA)-2) quaternization agent was added in a molar ratio CH_3_I to amines 2:1, in order to achieve 100% conversion of the tertiary amine group to quaternary ammonium salt. The reaction was carried out at room temperature for 24 h, under stirring and the absence of intense light due to methyl iodide light sensitivity. The excess of CH_3_I and THF were removed by rotary evaporator and vacuum drying at room temperature for 48 h. The modification procedure followed for the production of quaternized P(QDMAEMA-co-OEGMA) copolymers is also depicted in Scheme 1.

### 2.3. Size Exclusion Chromatography (SEC)

The molar masses (M_w_), molar mass distributions, and polydispersity index (M_w_/M_n_) of the synthesized P(DMAEMA-co-OEGMA) copolymers were determined by SEC, using a Waters instrument (Waters Corporation, Milford, MA, USA). The chromatography system consists of a Waters 1515 isocratic pump (Waters Corporatione, Milford, MA, USA), a set of three m-Styragel mixed pore separation columns (pore size 10^2^–10^6^ Å), and a Waters 2414 differential refractive index detector (equilibrated at 40 °C) (Waters Corporation, Milford, MA, USA). The measurements and data analysis were performed using the Breeze software (version 3.2) (Waters Corporation, Milford, MA, USA). Tetrahydrofuran containing 5% *v*/*v* trimethylamine was the mobile phase, at a flow rate of 1 mL/min, and the temperature was set at 30 °C. Linear polystyrene standards with average molecular mass in the range of 1200–929,000 g·mol^−1^ and narrow molecular mass distributions were utilized for setting up the calibration curve. P(DMAEMA-co-OEGMA) copolymers were soluble in the mobile phase and were measured at concentrations in the range of 2–4 mg mL^−1^.

### 2.4. Proton Nuclear Magnetic Resonance (^1^H-NMR) Spectroscopy

^1^H-NMR experiments were conducted to confirm the chemical structure and to determine the mass composition (%wt.) of the synthesized copolymers, as well as their quaternized derivatives. The measurements were operated on a Bruker AC 300 MHz FT-NMR spectrometer (Bruker, Billerica, MA, USA) using deuterium oxide (D_2_O) as the deuterated solvent. Tetramethylsilane (TMS) was utilized as the internal standard and the chemical shifts of the recorded spectra are reported in parts per million (ppm).

^1^H-NMR spectral peaks of P(DMAEMA-co-OEGMA) copolymers (300 MHz, D_2_O, ppm): 4.16 (peak c: 2H, -OCH_2_CH_2_N-, 2H, -(C=O)OCH_2_CH_2_O), 3.66 (peak f: 4H, -(CH_2_CH_2_O)_19_CH_3_-), 3.35 (peak g: 3H, -(CH_2_CH_2_O)_19_CH_3_-), 2.74 (peak d: 2H, -OCH_2_CH_2_N-), 2.32 (peak e: 3H,-N(CH_3_)_2_), 1.91 (peak b: 2H, -CH_2_C-), 0.90 (peak a: 3H, -CH_2_CCH_3_-).

^1^H-NMR spectral peaks of P(QDMAEMA-co-OEGMA copolymers (300 MHz, D_2_O, ppm): 4.54 (peak c_1_: 2H, -OCH_2_CH_2_N-), 4.22 (peak c_2_: 2H, -(C=O)OCH_2_CH_2_O)), 3.68 (peak f: 4H, -(CH_2_CH_2_O)_19_CH_3_-), 3.32 (peak e: 9H, -N(CH_3_)_3_) and peak g: 3H,-(CH_2_CH_2_O)_19_CH_3_-)), 3.05 (peak d: 2H, -OCH_2_CH_2_N), 2.07 (peak b: 2H, -CH_2_C-), 1.05 (peak a: 3H, -CH_2_CCH_3_-).

### 2.5. Fourier-Transform Infrared (FT-IR) Spectroscopy

Fourier-transform infrared spectroscopy (FTIR) measurements were performed in order to verify the chemical structure of the P(DMAEMA-co-OEGMA) copolymers and particularly to certify the modification of the tertiary amines of the precursor copolymers to quaternized ammonium salts. The measurements were implemented at room temperature in the range of 5000–550 cm^−1^ using a Fourier transform instrument (Bruker Equinox 55, Bruker Optics GmbH, Ettlingen, Germany) equipped with a single bounce attenuated total reflectance (ATR) diamond accessory (Dura-Samp1IR II by SensIR Technologies, Chapel Hill, NC, USA). The copolymer samples were analyzed in the solid state and the spectra were recorded after 64 scans with a resolution of 4 cm^−1^.

ATR-FTIR spectral peaks of P(DMAEMA-co-OEGMA), *v* (cm^−1^), (s: stretching, b: bending): (CH_2_): 2922 (s), 2854 (s) and 1458 (b), (-N(CH_3_)_2_): 2821 (s) and 2765 (s), (C=O): 1722 (s), (C-N): 1145 (s), (-O=C-O-C): 1100 (s).

ATR-FTIR spectral peaks of P(QDMAEMA-co-OEGMA), *v* (cm^−1^): (O-H) (internal moisture) 3467 (s) and 1640 (b), (C-N(CH_3_)_3_^+^): 3005 (s), 1474 (s) and 950 (s), (CH_2_): 2871 (s) and 1458 (b), (C=O): 1722, (C-N): 1145 (s), (-O=C-O-C): 1100 (s).

### 2.6. Cell Culture and Exposure to Copolymer Aggregates

HEK293, 4T1, MDA-MB-231, MCF-7, and T47D cell lines were grown in DMEM high glucose culture medium that contained 10% FBS, 2 mmol/L glutamine, 100 ΙU/mL of penicillin, and 100 μg/mL streptomycin at 37 °C. Every 48 h, the medium was replaced and cells were passaged on a weekly basis using the typical trypsin-EDTA concentrations. All cell lines were incubated for approximately 24 h at 37 °C with copolymer aggregates at concentrations that ranged from 0.5 to 150 μg/mL. MDA-MB-231, MCF-7, and T47D are human breast cancer cell lines that are either triple negative (MDA-MB-231) [39] or estrogen and progesterone receptor-positive (MCF-7 and T47D) [40,41]. 4T1 is a P53 null and highly metastatic cell line derived from mice [42] and HEK293 (human embryonic kidney) cells were used as a control cell line (non-cancerous) [43]. For testing in vitro cytotoxicity of the double hydrophilic P(DMAEMA-co-OEGMA) copolymers and their quaternized analogs, aqueous solutions of the copolymers at a concentration of 1 mg mL^−1^ were prepared using sterile water.

### 2.7. Estimation of Cell Viability (MTS Assay)

MTS assay (CellTiter 96^®^ Aqueous One Solution, Promega, Madison, WI, USA) was used to quantify the viability of cells exposed to the copolymer aggregates used. In this assay, the yellow tetrazolium [(3(4,5dimethylthiazolyl-2-yl)-5-(3-carboxymethoxyphenyl)-2-(4-sulfophenyl)-2H-tetrazolium), is reduced to formazan and the quantity of the latter is proportional to the number of living cells [44]. For this assay, a 96-well plate (corning-Costar, Corning, NY, USA) was used containing 5000 cells/well. Three types of controls were used: a positive (cells with culture medium were not exposed to copolymer aggregates), a negative (copolymer aggregates without cells), and a background control (culture medium alone). Cells that were incubated with copolymer aggregates for 24 h were rinsed once and 100 μL of serum-free medium and 20 μL of MTS reagent were added according to the manufacturer’s instructions. After an incubation time of 2 h, the plate was read at 490 nm using a microplate spectrophotometer (SPECTROstarNano, BMG LABTECH, Ortenberg, Germany).

### 2.8. Formation of Polyplexes

The double hydrophilic P(DMAEMA-co-OEGMA) and P(QDMAEMA-co-OEGMA) copolymers in aqueous milieu form nanosized aggregates. Aqueous solutions of the copolymers (1 mg mL^−1^) and DNAs (0.94 mg mL^−1^ for P(DMAEMA-co-OEGMA)-1, 0.66 mg mL^−1^ for P(QDMAEMA-co-OEGMA)-1 and 0.55 mg mL^−1^ for P(QDMAEMA-co-OEGMA)-2) were prepared in NaCl 0.01 M at ambient temperature and neutral pH (pH = 7). The concentrations of copolymers and DNAs solutions used are based upon calculations related to the positive charges of the amino groups (N) of DMAEMA/QDMAEMA and the negative charges of DNA phosphate groups (P). Complexation was achieved by mixing the solutions of copolymer aggregates and DNAs. The electrostatic interactions between the amino groups of copolymers and phosphates groups of DNAs lead to the formation of polyplexes in N/P ratio generally ranging from 0.25 to 8. Mixing was carried out by retaining a constant volume of each copolymer solution (2 mL) and by adding into it the appropriate amount (depending on the desired charge ratio) of DNA, under gentle stirring, at room temperature, and pH = 7. The mixed copolymer aggregates/DNA solutions were incubated at room temperature overnight for the equilibration of the formed polyplexes.

### 2.9. Fluorescence Spectroscopy-Ethidium Bromide Quenching Assay

The binding affinity of P(DMAEMA-co-OEGMA) and P(QDMAEMA-co-OEGMA) copolymer aggregates to DNAs was examined by fluorescence spectroscopy (FS) through the quenching of ethidium bromide (EtBr). DNA solutions of 113 bp and 2000 bp (0.1 mg mL^−1^) were prepared in NaCl 0.01 M. Then, ethidium bromide was added in the DNA solutions at a molar ratio, [EtBr] = [P]/4, where [P] corresponds to the molar concentration of DNA phosphate groups. The DNA solutions containing EtBr were left overnight to equilibrate. Similarly, concentrated solutions of copolymer aggregates (1 mg mL^−1^) were also prepared in NaCl 0.01 M. The labeled DNA-EtBr solutions were titrated using copolymer aggregates solutions, in the range of N/P ratio from 0 (neat DNA-EtBr solution) to 8. The titration was followed by fluorescence spectroscopy. The measurements were conducted on a Fluorolog-3 Jobin Yvon-Spex spectrofluorometer (model GL3–21) (Kyoto, Japan). The solutions of polyplexes were equilibrated for 15 min at 25 °C before FS measurements. The excitation wavelength used for the recorded spectra was at 535 nm, while the emission was monitored at 600 nm.

### 2.10. Ultraviolet–Visible (UV–Vis) Spectroscopy

The complexation ability of the P(DMAEMA-co-OEGMA) and P(QDMAEMA-co-OEGMA) copolymer aggregates with DNAs was further investigated by ultraviolet–visible (UV–vis) spectroscopy. The copolymer aggregates/DNAs were measured at different N/P ratios and their absorption spectra were recorded on a Perkin Elmer (Lambda 19) UV–vis–NIR spectrophotometer (Waltham, MA, USA) in the range of 200–400 nm.

### 2.11. Light Scattering

Light scattering studies were conducted to determine the size, surface charge, and morphology of the formed polyplexes. Dynamic light scattering (DLS) measurements were performed on an ALV/CGS-3 compact goniometer system (obtained from ALV GmbH, Langen, Hessen, Germany), equipped with a JDS Uniphase 22 mW He–Ne laser (ALV GmbH, Langen, Hessen, Germany as a light source, operating at 632.8 nm, interfaced with an ALV-5000/EPP multi-τ digital correlator (ALV GmbH, Langen, Hessen, Germany) with 288 channels and an ALV/LSE-5003 light scattering electronics (ALV GmbH, Langen, Hessen, Germany) unit for stepper motor drive and limit switch control. Toluene was used as the calibration standard. The solutions were loaded into cylindrical optical glass cuvettes (obtained from LS Instruments, Fribourg, Switzerland) and measurements were conducted at an angular range of 45° to 135°, at 25 °C. Five measurements were executed for each angle and were averaged. The autocorrelation functions were fitted and analyzed by the cumulants method and the CONTIN algorithm. The presented data of hydrodynamic radius (R_h_) and light scattering intensity (I) correspond to measurements at 90°. Before measurements, all the solutions were filtered through 0.45 μm hydrophilic PVDF syringe filters (obtained from Membrane Solutions, Auburn, WA, USA) to remove large aggregates and dust particles and they were equilibrated for 15 min at 25 °C.

Static light scattering (SLS) measurements were performed on the same instrument in the angular range of 30–150°, using toluene as the calibration standard. SLS measurements were treated by the Zimm and Guinier plots in order to estimate the R_g_/R_ho_ ratios after extrapolation to zero angle.

Electrophoretic light scattering (ELS) measurements were also performed at 25 °C using a Nano Zeta Sizer (Malvern Instruments Ltd., Worcestershire, UK) composed of a 4 mW solid-state He–Ne laser, operating at 633 nm and at a fixed backscattering angle of 173°. Zeta-potential values were determined using the Henry correction of the Smoluchowski equation.

## 3. Results and Discussion

### 3.1. Synthesis and Characterization of Random Copolymers

**Figure 1:**
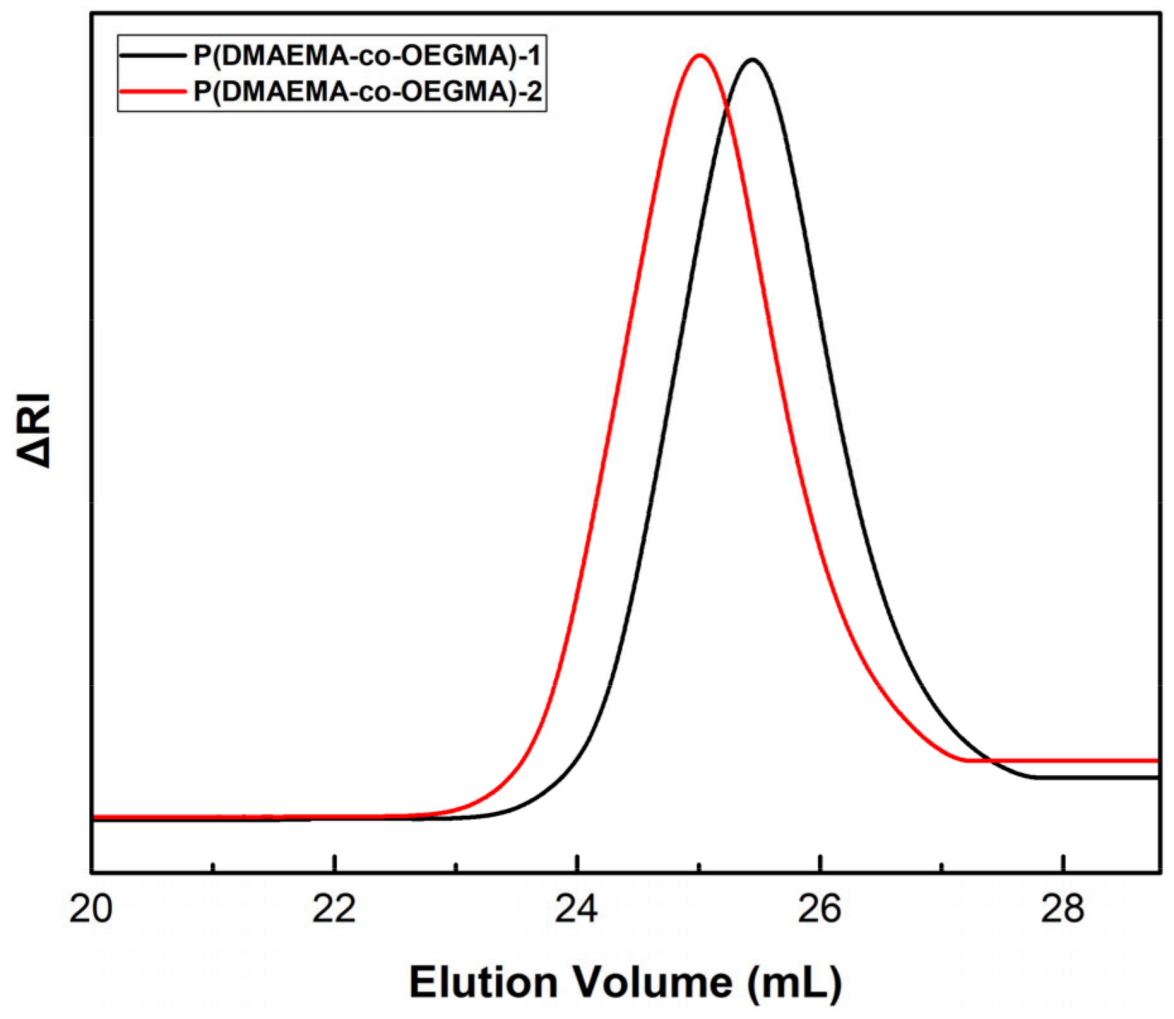
SEC chromatograms of the synthesized P(DMAEMA-co-OEGMA)-1 and P(DMAEMA-co-OEGMA)-2 copolymers in THF/5% *v*/*v* Et_3_N.

Two poly[(2-(dimethylamino)ethyl methacrylate)-co-oligo(ethylene glycol) methyl ether methacrylate] P(DMAEMA-co-OEGMA) double hydrophilic copolymers were synthesized in a facile way via RAFT polymerization following a one-step synthetic procedure (Scheme 1). The copolymers were synthesized in different molar masses and compositions of the two segments, utilizing the oligomer OEGMA with average M_n_ = 950 g·mol^−1^ and 19 repeated units of ethylene glycol. 4-Cyano-4-[(dodecyl sulfanylthio carbonyl)sulfanyl]pentanoic acid (CPD) was selected as the chain transfer agent for the synthesis of these copolymers, since it is known from the literature that it is reactive and well-suitable for methacrylate monomers [37]. Moreover, it has been also utilized in our previous works for the polymerization of similar methacrylate monomers and resulted in well-defined polymers and controlled polymerization processes [27,31]. The copolymers were molecularly characterized by SEC and the determined molar masses and polydispersity index (M_w_/M_n_) values are listed in Table 1. The resulting molar masses are close to the stoichiometry and the M_w_/M_n_ values are in a satisfactory range for RAFT polymerization procedures. The chromatograms of both P(DMAEMA-co-OEGMA) copolymers as obtained by SEC, which are presented in Figure 1, show the synthesis of well-defined copolymers with relatively narrow, monomodal, and symmetric molar mass distributions.

The P(DMAEMA-co-OEGMA) copolymers were further chemically modified by the quaternization of the tertiary amine group of the DMAEMA segment to quaternary ammonium salt. The followed modification procedure as well as the chemical structure of the quaternized P(QDMAEMA-co-OEGMA) derivatives are also depicted in Scheme 1. The quaternized derivatives were transformed to strong cationic polyelectrolytes with the QDMAEMA segment carrying a permanent positive charge, capable of exhibiting higher binding affinity to nucleic acids.

**Figure 2:**
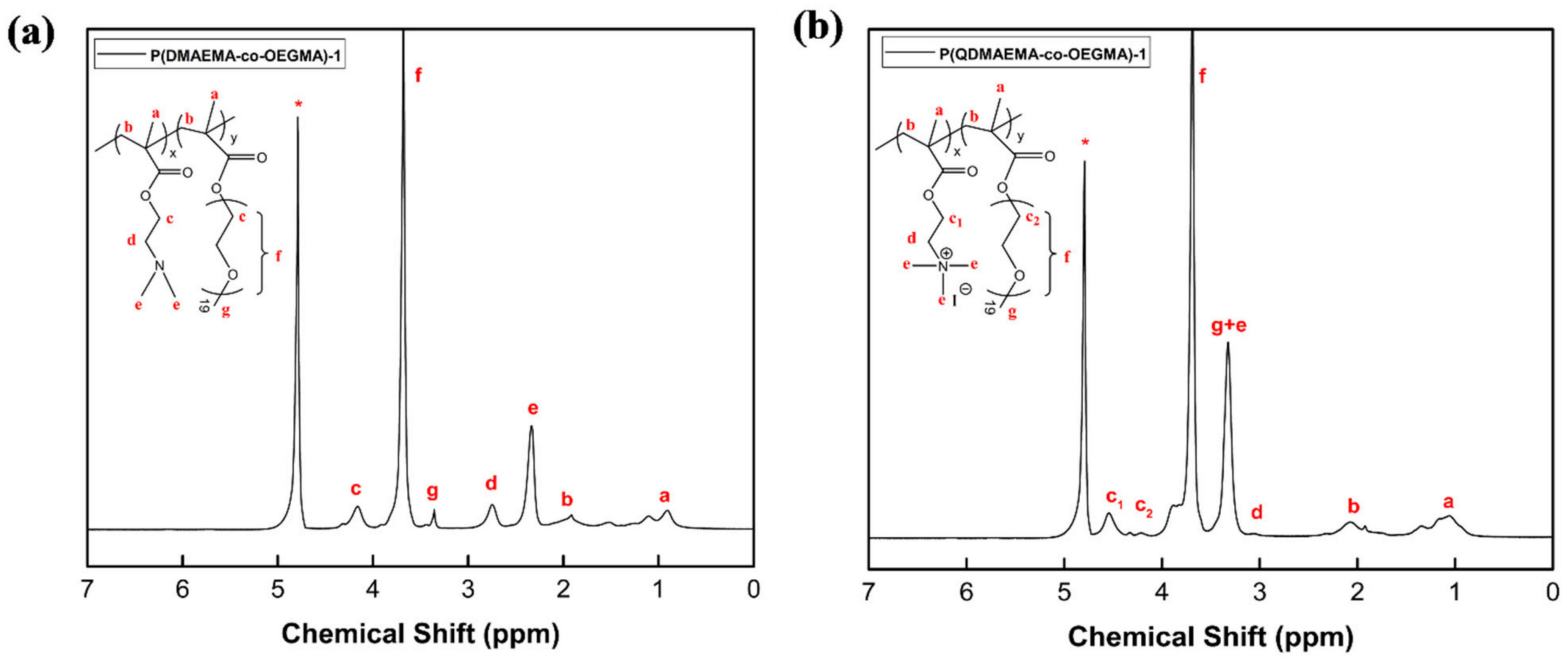
Representative ^1^H-NMR spectra of (**a**) P(DMAEMA-co-OEGMA)-1 and (**b**) P(QDMAEMA-co-OEGMA)-1 copolymers in D_2_O. The asterisk at 4.79 ppm denotes the peak of the solvent.

The chemical structure and the composition of the synthesized copolymers were estimated by ^1^H-NMR spectroscopy. Spectra analysis confirmed the expected structure of the synthesized precursor copolymers as well as the successful completion of the quaternization reaction. Representative spectra of P(DMAEMA-co-OEGMA)-1 and P(QDMAEMA-co-OEGMA)-1 copolymers are provided in Figure 2. The composition of P(DMAEMA-co-OEGMA) copolymers (Figure 2a) was evaluated by integrating the characteristic spectral peak at 2.32 ppm of the -CH_3_ protons of the DMAEMA amino group (peak e, 6H, -N(C*H_3_*)_2_) and the -CH_2_ protons at 3.66 ppm of the OEGMA ethylene glycol side chain (peak f, 4H, -(C*H*_2_C*H_2_*O)_19_CH_3_-) [27]. According to the obtained results, mass composition values of the P(DMAEMA-co-OEGMA) copolymers were very close to the stoichiometric ones and are also included in Table 1. Concerning P(QDMAEMA-co-OEGMA) copolymers (Figure 2b), the signals of -CH_3_ protons of the quaternary amine are expected to be detected at approximately 3.32 ppm (peak e, 9H, -N(CH_3_)_3_) [45]. However, in the same spectral region the -CH_3_- protons of OEGMA (peak g, 3H, -CH_3_) are also detected. Hence, there is an overlap of the signals of the peaks e and g. Moreover, because of the low quality of the spectra of the quaternized copolymers due to aggregation phenomena and the overlap of the characteristic peaks, it was difficult to estimate the accurate mass compositions based on ^1^H-NMR spectroscopy. Therefore, in the case of the quaternized copolymers, ^1^H-NMR spectroscopy was utilized in a qualitative base, in order to detect structural changes after the quaternization reaction. Nevertheless, the absence of the peak assigned to the methyl protons of the tertiary amine group at 2.32 ppm in Figure 2b compared with the peak e of the precursor (Figure 2a) and the shift to 3.32 ppm despite the overlap indicate that the quaternization reaction was, in fact, quantitative and the tertiary amine group of the DMAEMA segment was successfully converted to quaternary ammonium salt. Hence, the composition and molar masses of the quaternized copolymers were calculated according to the molecular characteristics of the precursor P(DMAEMA-co-OEGMA) copolymers and also considering that quaternization reaction is quantitative [45,46] and the conversion of the tertiary to quaternary amines occurs at 100%. The estimated molecular characteristics of quaternized copolymers are presented in Table 1.

**Figure 3:**
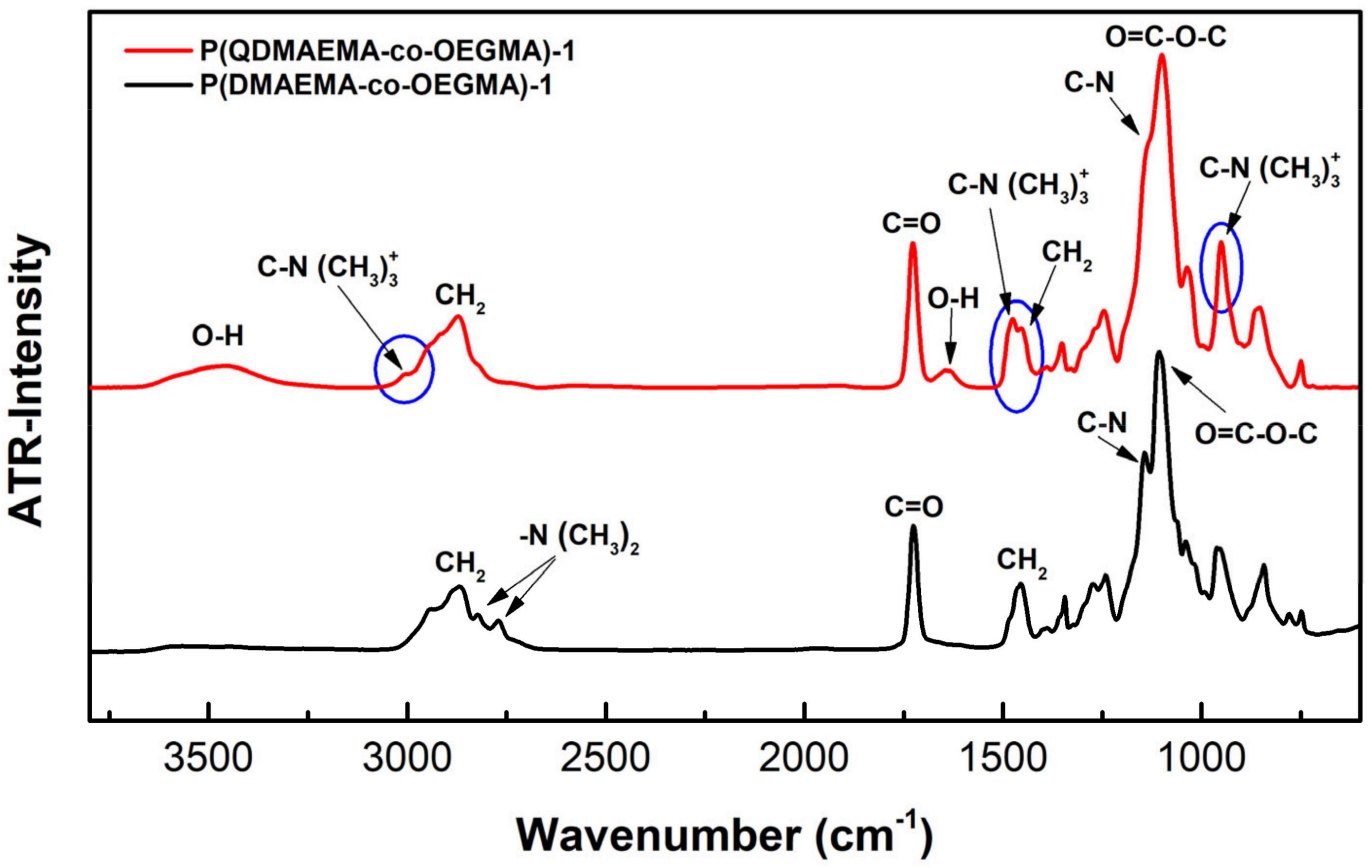
Representative ATR-FTIR spectra of P(DMAEMA-co-OEGMA)-1 and P(QDMAEMA-co-OEGMA)-1 copolymers in solid state.

Additionally, FTIR spectroscopy was also implemented to certify the chemical structure of the P(DMAEMA-co-OEGMA) copolymers, and especially to further confirm the conversion of the tertiary amine group to quaternary ammonium salt. ATR-FTIR spectra of P(DMAEMA-co-OEGMA)-1 and its quaternized analog are presented in Figure 3. The spectral fingerprint of P(DMAEMA-co-OEGMA)-1 copolymer contains all the characteristic bands that certify the expected chemical structure. Concerning the P(QDMAEMA-co-POEGMA)-1 and comparatively with its precursor, the disappearance of the characteristic bands of the tertiary amines N(CH_3_)_2_ at 2820 cm^−1^ and 2770 cm^−1^, as well as the presence of new absorption peaks at 3005 cm^−1^, 1474 cm^−1^, and 950 cm^−1^ corresponding to quaternary amine groups [31,47], verified the quaternization modification.

### 3.2. In Vitro Cytotoxicity of Random Copolymer Aggregates

**Figure 4:**
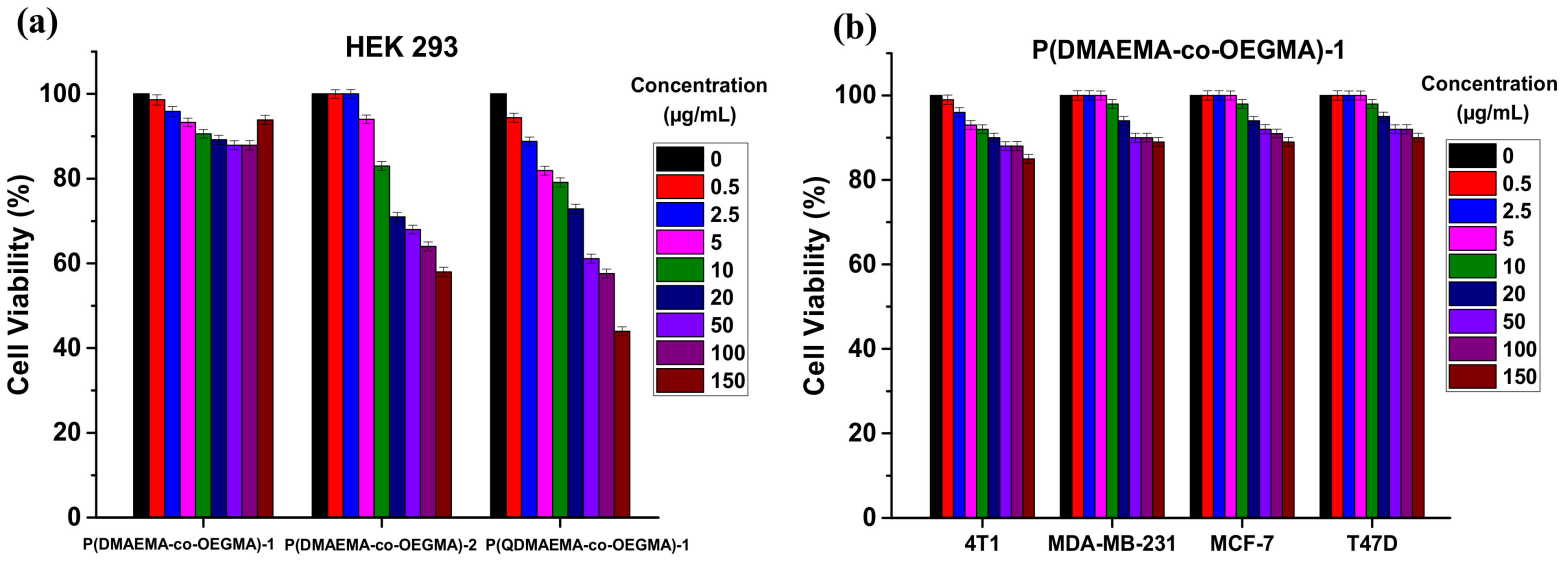
(**a**) MTS cell viability assay after 24 h treatment of HEK293 with various concentrations of P(DMAEMA-co-OEGMA)-1, P(DMAEMA-co-OEGMA)-2, and P(QDMAEMA-co-OEGMA)-1 copolymer aggregates. (**b**) MTS cell viability assay after 24 h treatment of 4T1, MDA-MB-231, MC-7, and T47D with various concentrations of P(DMAEMA-co-OEGMA)-1 copolymer aggregates. The concentration of 0 μg/mL corresponds to the untreated cells. Cell viability is expressed as % of cell viability ± SD between two experiments.

The cytotoxicity effect of non-viral polymeric-based vectors and especially of PDMAEMA remains a significant drawback for their implementation in gene delivery. Hence, the biocompatibility of cationic polymers is an aspect of great importance, which should be considered in the design of effective gene delivery nanocarriers. In this regard, the cytotoxicity of P(DMAEMA-co-OEGMA) and P(QDMAEMA-co-OEGMA) copolymer aggregates was assessed in the human embryonic kidney cell line (HEK293) and the cell viability was evaluated by the MTS assay. Figure 4a demonstrates the percentage of the viable HEK293 cells after their exposure to increasing concentrations of the P(DMAEMA-co-OEGMA)-1, P(DMAEMA-co-OEGMA)-2 and P(QDMAEMA-co-OEGMA)-1 copolymer aggregates. Based on MTS assay, HEK293 cells treated with P(DMAEMA-co-OEGMA)-1 copolymer aggregates present a high percentage of viability (>88%) in the whole range of the concentrations (0.5–150 μg/mL) tested. On the contrary, P(DMAEMA-co-OEGMA)-2 and P(QDMAEMA-co-OEGMA)-1 copolymer aggregates exhibit a dose-dependent cytotoxicity on HEK293 cells, with almost half of the cells exposed to the concentration of 150 μg/mL remaining viable. Particularly, 54% of the cells are viable when treated with P(DMAEMA-co-OEGMA)-2 and 44% with P(QDMAEMA-co-OEGMA)-1 copolymer aggregates. Nevertheless, at lower concentrations and until the one at 10 μg/mL, the % cell viability is approximately 80% for both copolymer aggregates, which is a tolerable level. However, the toxicity of P(DMAEMA-co-OEGMA)-2 copolymer aggregates was not expected. In this copolymer, the prevalence of the OEGMA segment according to its larger mass ratio (wt. 65%) was expected to provide more biocompatibility and stealth properties to the aggregates. On the contrary, the aggregates of P(DMAEMA-co-OEGMA)-1 copolymer that consisted of a lower content of OEGMA segments (wt. 54%) presented enhanced cell viability compared with the P(DMAEMA-co-OEGMA)-2 copolymer aggregates. This fact can be assigned to differences related to molecular characteristics of the copolymers (actual segment distribution along the chain, molar mass, and dispersity) and solution properties of the aggregates.

On the other hand, the cytotoxicity of P(QDMAEMA-co-OEGMA)-1 copolymer aggregates was quite expected. Similar cytotoxicity behavior was exhibited by analogous quaternized polymeric systems studied in our previous work [27]. It is known that the cytotoxicity of polycations is related to the polyamine nature (i.e., primary, secondary, tertiary, and quaternary amino groups) [14]. Hence, the enhanced toxicity of these copolymer aggregates is probably resulting from the permanent positive charges of the quaternary amino groups. Consequently, it is evident that the nature of the amino group impacts on the biocompatibility profile of these polymeric systems. Moreover, it is noteworthy to note the effect of the OEGMA segment in the biocompatibility performance, especially of the P(DMAEMA-co-OEGMA)-1 polymeric system. The utilization of the OEGMA with 19 ethylene glycol repeated units seems to ameliorate cytotoxicity effects, due to its enhanced stealth and shielding properties. Furthermore, the percentage (%) of cell viability remains at high levels at the studied concentrations, compared with the similar PDMAEMA-b-POEGMA polymeric system with OEGMA of nine ethylene glycol repeated units, which was studied in our previous work [27]. In that case, the percentage (%) of cell viability was found to decrease with increasing concentration. However, the direct comparison between these systems is not easy because there are several factors, such as chain architecture, molar mass, composition of the copolymers, and surface charge of the aggregates that influence their cytotoxicity behavior.

The encouraging cytotoxicity results of P(DMAEMA-co-OEGMA)-1 copolymer aggregates led to further investigation of their biocompatibility profile on cancerous cell lines. Particularly, their cytotoxicity was evaluated toward three breast cancer cell lines including the metastatic 4T1 cell line derived from mice, the triple-negative MDA-MB-231 cell line, and also the estrogen and progesterone receptor-positive MCF-7 and T47D cell lines derived from human breast carcinoma. Our future plans involve the utilization of these polymeric systems for the interaction and delivery of a specific miRNA associated with breast cancer and triple-negative breast cancer. The evaluation of their in vitro biological performance will be implemented using the above-mentioned human breast cancer cell lines. Hence, the cytotoxicity of P(DMAEMA-co-OEGMA)-1 copolymer aggregates was selected to be examined at the above-mentioned cell lines, mainly to ensure that the empty nanocarriers do not provoke cytotoxic activity. Indeed, according to the results presented in Figure 4b, copolymer aggregates do not exhibit cytotoxicity. The percentage (%) of cell viability remains at a high level at the whole concentration range. Indicatively, the determined % of cell viability at the maximum concentration was 85% of the 4T1 cells and approximately 90% of the cells of the other three human breast cancer cell lines. In summary, the overall cytotoxicity findings of P(DMAEMA-co-OEGMA)-1 copolymer aggregates evaluated at cancerous and non-cancerous cell lines demonstrated their high biocompatibility. Moreover, according to the in vitro results, this polymeric system can be characterized as non-toxic, providing the potential for its further biological application.

### 3.3. Ethidium Bromide Quenching Assay by Fluorescence Spectroscopy

Ethidium bromide (EtBr) was utilized as a fluorescent probe for studying the interaction ability of P(DMAEMA-co-OEGMA) and P(QDMAEMA-co-OEGMA) copolymer aggregates with DNAs of 113 bp and 2000 bp. The cationic nature of EtBr compound permits the electrostatic binding to double-stranded DNA molecules by its intercalation into the base pairs of the double helix [48,49]. Moreover, the intercalated EtBr into DNA displays strong fluorescence intensity. The interaction of the cationic polymer with the DNA-EtBr complex results in a competitive exclusion of the intercalated EtBr from the DNA double helix [48]. The displacement of EtBr from the double helix of DNA to the solution is monitored as a decrease in its fluorescence intensity. Therefore, the quenching of EtBr indicates the binding affinity of the cationic polymer by forming polyplexes with DNA.

**Figure 5:**
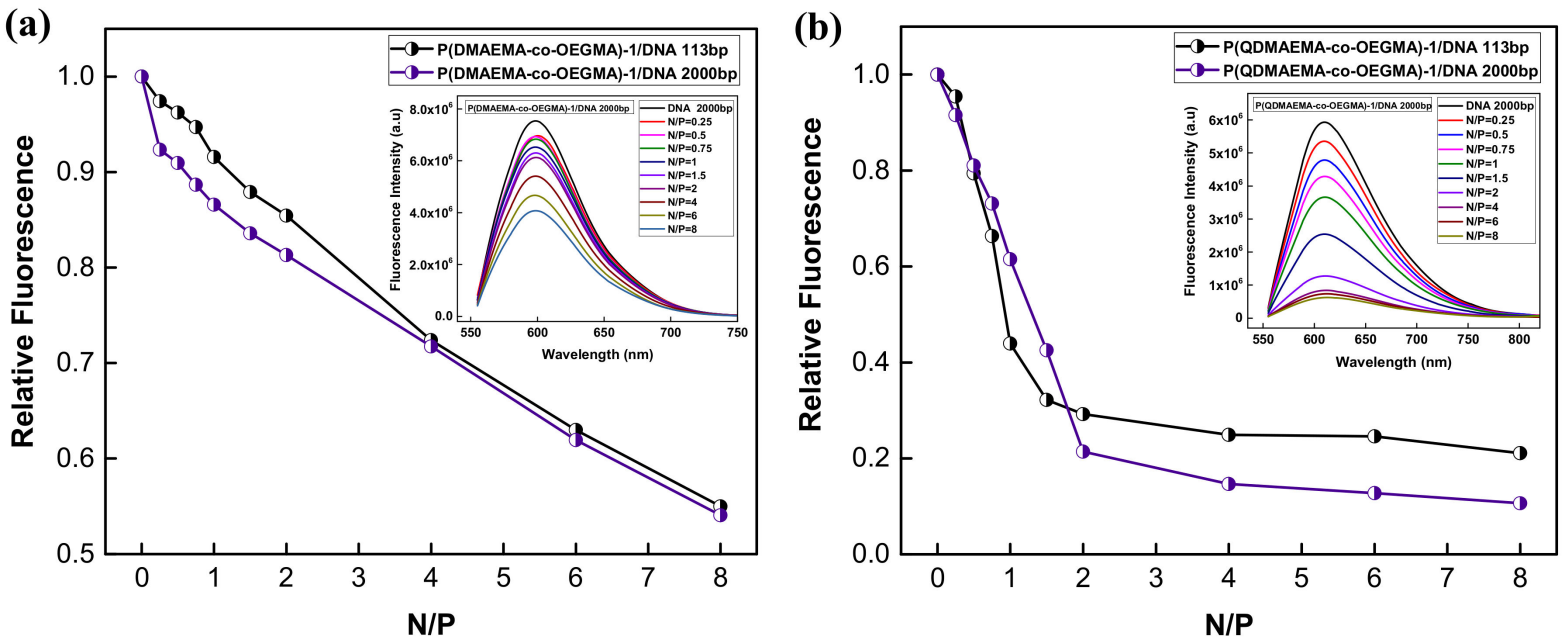
Ethidium bromide fluorescence quenching in (**a**) P(DMAEMA-co-OEGMA)-1/DNA of 113 bp and 2000 bp and (**b**) P(QDMAEMA-co-OEGMA)-1/DNA of 113 bp and 2000 bp polyplexes. Indicative fluorescence spectra of the intercalated ethidium bromide for polyplexes with DNA of 2000 bp are also included in Figure 5a,b.

In this regard, the quenching of EtBr was investigated in a range of N/P = 0 to N/P = 8 ratio, by titration of copolymer solutions to the pretreated DNA-EtBr solution, followed by recording of the fluorescence intensity. The relative fluorescence intensity of the intercalated EtBr upon increasing N/P ratio is depicted in Figure 5a,b for P(DMAEMA-co-OEGMA)-1/DNAs and P(QDMAEMA-co-OEGMA)-1/DNAs polyplexes. Moreover, representative spectra showing the reduction in the fluorescence intensity of EtBr at the studied N/P ratio range are also included in Figure 5a,b. These spectra are indicative of the decrease in EtBr in the solutions of the copolymer aggregates with the DNA of 2000 bp. Hence, the reduction in fluorescence intensity of the recorded spectra against the increase in the N/P ratio is reflected in the relative fluorescence intensity, indicating the displacement rate of EtBr in the solutions of copolymer aggregates/DNAs. Moreover, the displacement of EtBr is evident in both types of copolymer/DNA complexes. Particularly, in Figure 5a the decrease in the relative fluorescence of EtBr is obvious during the interaction of P(DMAEMA-co-OEGMA)-1 copolymer aggregates with both DNAs. However, the copolymer aggregates interacting either with DNA 113 bp or DNA 2000 bp present a similar trend in the decrease in relative fluorescence intensity, and in the case of DNA 2000 bp is slightly steeper. Therefore, the displacement of EtBr from its complexes with the DNA of 2000 bp is a bit faster compared with the DNA of 113 bp, implying a better complexation ability of the P(DMAEMA-co-OEGMA)-1 with the DNA of 2000 bp. Nevertheless, the differences are small, since they present a sharp decrease in the relative intensity of EtBr in the range N/P = 2 to N/P = 8 in which the ratio of the relative intensity of the displacement of the intercalated EtBr reaches 0.55 for the polyplexes with the DNA 113 bp and 0.54 for those with the DNA 2000 bp.

In the case of P(QDMAEMA-co-OEGMA)-1 copolymer aggregates with DNAs, the fluorescence spectroscopy results are quite different to those of P(DMAEMA-co-OEGMA)-1/DNAs polyplexes, as depicted in Figure 5b. Herein, the curves exhibit a well-pronounced and gradual decrease in the fluorescence intensity. However, this decrease is more abrupt for the polyplexes with the DNA of 2000 bp, where the expulsion of the intercalated EtBr occurs in a faster rate up to N/P = 2 in comparison with the polyplexes prepared with the DNA of 113 bp whose decrease is gradual and slower until the same N/P ratio. The relative fluorescence intensity of EtBr reaches a plateau from N/P = 2 to N/P = 8, for the polyplexes of both DNAs. Additionally, the relative fluorescence intensity at the ratio N/P = 8 was found to be equal to 0.21 for polyplexes of DNA of 133 bp and 0.10 for polyplexes of 2000 bp. Undoubtedly, the recorded displacement rates of EtBr fluorescence intensity demonstrate the strong binding affinity of the quaternized P(QDMAEMA-co-OEGMA)-1 copolymer aggregates with both DNAs, and slightly stronger with the DNA of 2000 bp.

In summary, the ethidium bromide quenching assay confirmed the ability of both copolymer aggregates to efficiently bind DNAs and form polyplexes. The quaternized copolymer aggregates displayed better complexation ability probably due to the permanent cationic charge of the quaternary QDMAEMA amino group. On the contrary, the partially positively charged tertiary amino group of P(DMAEMA-co-OEGMA)-1 copolymer resulted in lower DNA binding efficiency. Another explanation for the low binding affinity of the P(DMAEMA-co-OEGMA)-1 copolymer aggregates is probably the shielding of the positive charges by the OEGMA chains. In the case of the quaternized copolymer aggregates, the shielding effect is maybe not so evident due to the fully cationic QDMAEMA chains. Therefore, the positive charge of the DMAEMA/QDMAEMA cationic segments, the chain length of OEGMA moieties, and the length of the DNA play an essential role in the complexation ability of these copolymer aggregates.

It should be noted that regarding the exclusion of the intercalated EtBr and the binding affinity of the P(DMAEMA-co-OEGMA)-2/DNAs and P(QDMAEMA-co-OEGMA)-2/DNAs polyplexes (data not shown), similar behaviors and trends were observed.

### 3.4. Polyplexes Absorption Spectra by UV-Vis Spectroscopy

UV–vis spectroscopy was employed for further investigation on the electrostatic interaction between the copolymer aggregates and the DNAs of different lengths. The absorption of DNA molecules in the UV–vis spectral range allows the detection of alterations in the conformation of DNA chains, arising upon the interaction with cationic polymers. DNA spectrum presents a broad band in the range of 200–350 nm, with a λ_max_ at 260 nm [50,51]. During the complexation process with the positively charged copolymers and depending on the N/P ratio, the absorption intensity of this peak at 260 nm decreases and a new peak at shorter wavelength appears, approximately at 225 nm, corresponding to complexed DNA. It is noteworthy to mention that the copolymer aggregates do not display absorption peaks in the UV–vis region. These spectral changes of DNA imply the efficacious interaction with cationic copolymers and the formation of polyplexes.

**Figure 6:**
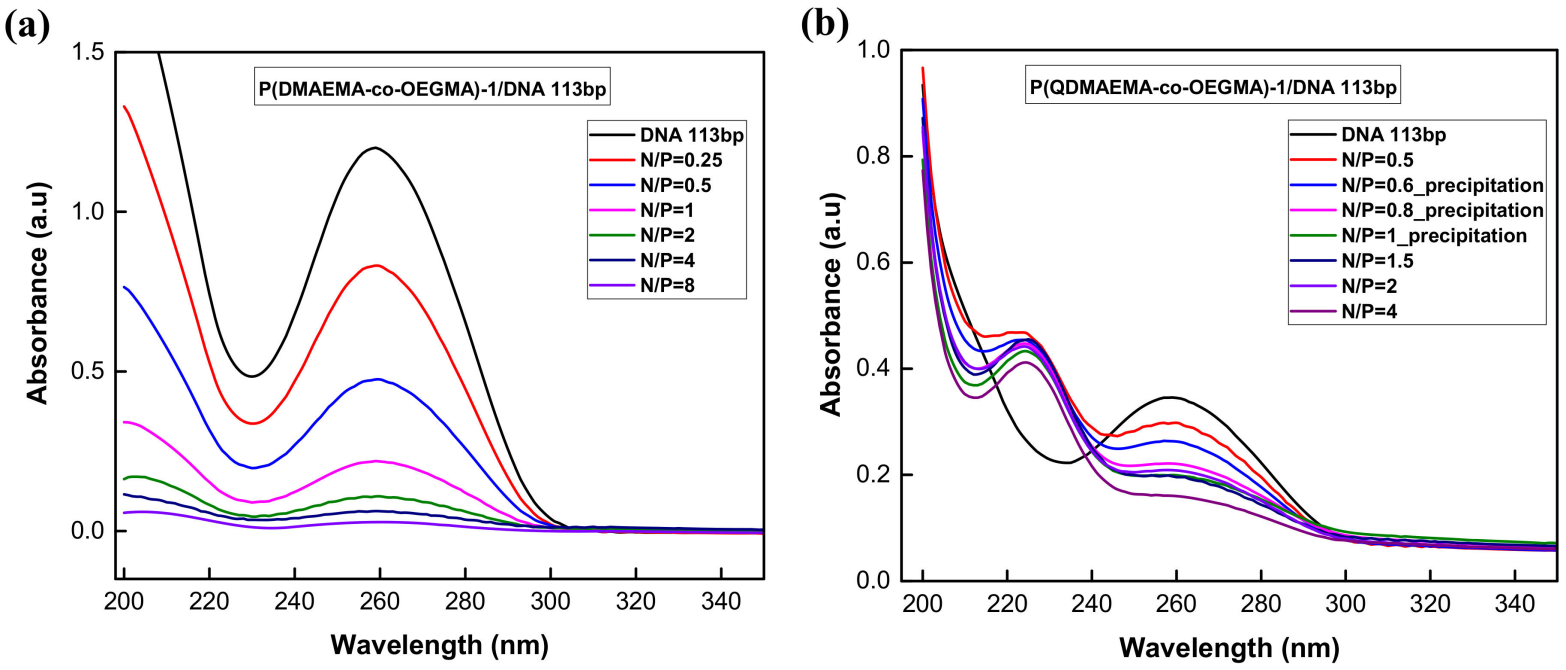
Representative UV–vis absorption spectra of (**a**) P(DMAEMA-co-OEGMA)-1/DNA 113 bp polyplexes and (**b**) P(QDMAEMA-co-OEGMA)-1/DNA 113 bp polyplexes in N/P ratios generally ranging from 0.25 to 8.

Representative absorption spectra of P(DMAEMA-co-OEGMA)-1/DNA 113 bp and P(QDMAEMA-co-OEGMA)-1/DNA 113 bp polyplexes are provided in Figure 6a,b. In the case of P(DMAEMA-co-OEGMA)-1/DNA 113 bp, the polyplexes were examined in different N/P ratios ranging from 0.25 to 8. The prepared aqueous solutions of P(DMAEMA-co-OEGMA)-1/DNA 113 bp polyplexes were colloidally stable and transparent in the whole N/P ratio range. Concerning the DNA spectral peaks, in Figure 6a the presence of only one peak at 260 nm is noticed, corresponding to free/non complexed DNA. The absorption intensity of this peak varies according to the N/P ratio, appearing more intense in N/P ratios below the neutralization ratio (N/P = 1), with an excess of phosphate groups. Nonetheless, the reduction in the absorption intensity of the DNA peak is also recorded at N/P ratios above N/P = 1 ratio, depicting the successful formation of polyplexes mainly at N/P ratios with an excess of amino groups. However, the electrostatic interactions of P(DMAEMA-co-OEGMA)-1 copolymer aggregates with DNA 113 bp are weak due to the partially positively charged DMAEMA segments.

On the other hand, regarding the interaction of P(QDMAEMA-co-OEGMA)-1 copolymer aggregates with DNA of 113 bp, the existence of two peaks at 260 nm and 225 nm is evident in Figure 6b, which are attributed to free/non complexed DNA and to complexed DNA, respectively. The aqueous solutions of P(QDMAEMA-co-OEGMA)-1/DNA 113 bp polyplexes were studied in N/P ratios ranging from 0.5 to 4. During the preparation of the polyplexes, the solutions appeared slightly opalescent. However, close to the neutralization point the polyplexes were partially precipitated at the N/P ratios of 1, 0.8, and 0.6. Thus, at these precipitated ratios UV–vis studies were performed by measuring the supernatant of the solutions. Herein, the peak at 225 nm is predominant at all the examined N/P ratios, even at those which showed precipitation. The strong absorption intensity of this peak demonstrates the successful interaction of copolymer aggregates with the DNA of 113 bp and the efficacious formation of polyplexes. However, the peak of free DNA at 260 nm is also apparent, with its absorption decaying upon increasing N/P ratio and gradually rising at lower N/P ratios. It is expected that at N/P ratios with excess of phosphate groups, only a part of the whole amount of the DNA molecules participates in the electrostatic interaction with the positively charged amino groups and therefore in the formation of the polyplexes, while the non-complexed DNA molecules are detected at 260 nm.

In conclusion, UV–vis spectroscopy confirmed the strong interaction of the P(QDMAEMA-co-OEGMA)-1 copolymer aggregates with DNA of 113 bp and the successful formation of polyplexes, despite the instability at certain ratios. In the case of P(DMAEMA-co-OEGMA)-1/DNA 113 bp polyplexes, UV–vis spectroscopy demonstrated their efficient formation but also revealed the weak interaction ability of P(DMAEMA-co-OEGMA)-1 with the DNA of 113 bp. A similar tendency was also observed for the polyplexes formed by the DNA of 2000 bp. Furthermore, similar results were obtained for P(DMAEMA-co-OEGMA)-2/DNAs and P(QDMAEMA-co-OEGMA)-2/DNAs polyplexes. The obtained UV–vis spectroscopy results are in compliance with the findings of fluorescence spectroscopy.

### 3.5. Light Scattering Studies on the Formed Polyplexes

**Figure 7:**
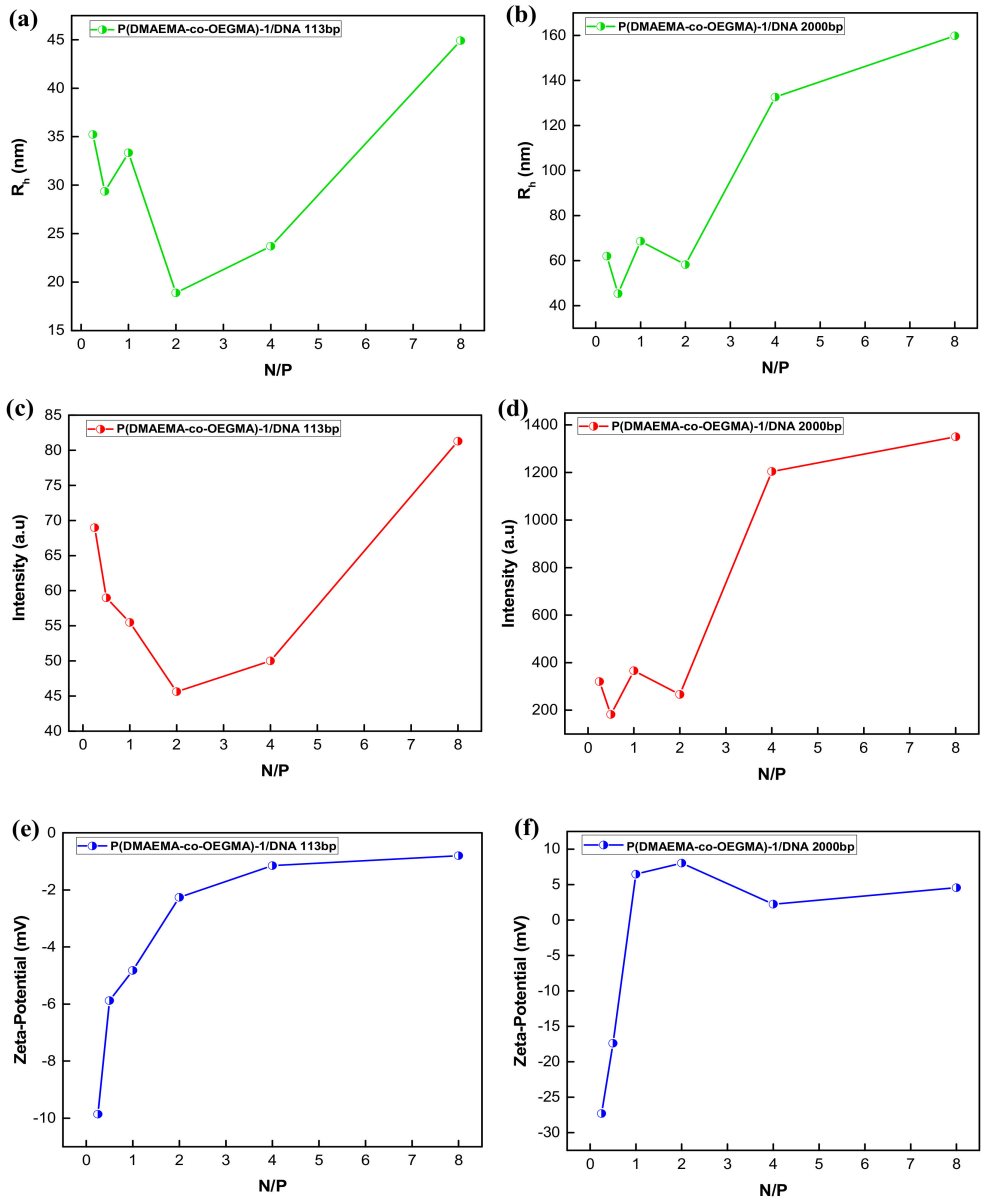
Variations of hydrodynamic radius (R_h_) with the N/P ratio for (**a**) P(DMAEMA-co-OEGMA)-1/DNA 113 bp and (**b**) P(DMAEMA-co-OEGMA)-1/DNA 2000 bp polyplexes. Variations of scattered light intensity with the N/P ratio for (**c**) P(DMAEMA-co-OEGMA)-1/DNA 113 bp and (**d**) P(DMAEMA-co-OEGMA)-1/DNA 2000 bp polyplexes. Variations of zeta potential with the N/P ratio for (**e**) P(DMAEMA-co-OEGMA)-1/DNA 113 bp and (**f**) P(DMAEMA-co-OEGMA)-1/DNA 2000 bp polyplexes.

**Figure 8:**
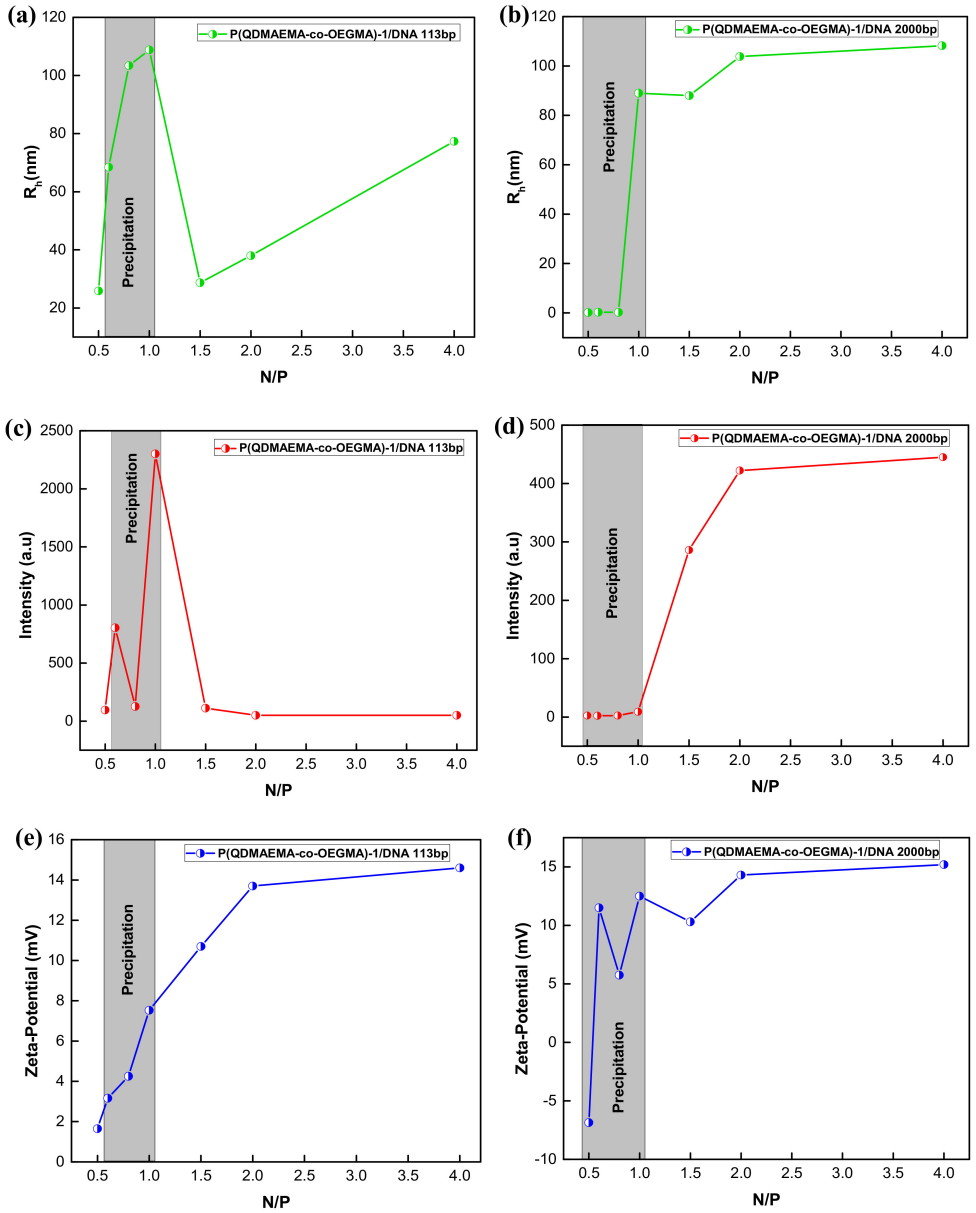
Variations of hydrodynamic radius (R_h_) with the N/P ratio for (**a**) P(QDMAEMA-co-OEGMA)-1/DNA 113 bp and (**b**) P(QDMAEMA-co-OEGMA)-1/DNA 2000 bp polyplexes. Variations of scattered light intensity with the N/P ratio for (**c**) P(QDMAEMA-co-OEGMA)-1/DNA 113 bp and (**d**) P(QDMAEMA-co-OEGMA)-1/DNA 2000 bp polyplexes. Variations of zeta potential with the N/P ratio for (**e**) P(QDMAEMA-co-OEGMA)-1/DNA 113 bp and (**f**) P(QDMAEMA-co-OEGMA)-1/DNA 2000 bp polyplexes.

The formed polyplexes resulting from the electrostatic interaction between the positively charged P(DMAEMA-co-OEGMA) and P(QDMAEMA-co-OEGMA) copolymer aggregates with the negatively charged DNAs of 113 bp and 2000 bp were studied by light scattering techniques. In particular, dynamic (DLS), electrophoretic (ELS), and static (SLS) light scattering were implemented to determine the size, surface charge (zeta potential), and the morphology of the formed polyplexes, respectively. The size and the surface charge of the formed polyplexes are fundamental parameters, determining their efficacy as non-viral gene delivery vectors. The polyplexes were studied in a wide range of N/P ratios, including ratios with an excess of amino groups of DMAEMA/QDMAEMA segments and an excess of DNAs phosphate groups, aiming to better understand the complexation process and to optimize the ratios with the better complexation efficiency and colloidal stability. Light scattering findings related to the size, intensity, and zeta potential of the polyplexes as a function of the N/P ratio are presented in Figure 7 for the P(DMAEMA-co-OEGMA)-1/DNAs and Figure 8 for the P(QDMAEMA-co-OEGMA)-1/DNAs polyplexes.

Before discussing the results based on the characteristics of the polyplexes as obtained by light scattering, it is noteworthy to present the sizes, surface charge, and the estimated R_g_/R_h_ ratio of the neat copolymer aggregates (empty nanovectors). P(DMAEMA-co-OEGMA)-1 copolymer in aqueous solution of NaCl 0.01 M formed nanosized aggregates with a size (R_h_) of 45 nm. Moreover, their surface charge was found approximately +4 mV, while the R_g_/R_h_ ratio acquired the value of 0.8, suggesting the formation of globular nanostructures. Similarly, the quaternized P(QDMAEMA-co-OEGMA)-1 copolymer formed aggregates in aqueous solutions of NaCl 0.01 M with R_h_ of about 70 nm, surface charge of +22 mV, and R_g_/R_h_ ratio of 0.9 also indicated globular structures but with the tendency to the formation of more elongated structures.

The strongly positive surface charge of the quaternized copolymer aggregates is attributed to the fully charged quaternary amine group. Whereas, the tertiary amines of precursor copolymer aggregates along with the long chain length of the non-ionic OEGMA segment resulted in less strong positive surface charge. Compared with our previous study [27] and relevant to the surface charge, the obtained zeta-potential values of PDMAEMA-b-POEGMA and QPDMAEMA-b-POEGMA were found to be +21.4 mV and +58.3 mV, respectively. Moreover, in this study the POEGMA with M_n_ = 475 g/mol and nine repeated ethylene glycol units was utilized for the production of the copolymers. Although, the polymeric systems present differences in their architecture, molar mass and other characteristics, the influence of the chain length of (P)OEGMA on the surface charge of the nanostructures is discernible. Therefore, judging from the zeta-potential values of the PDMAEMA-b-POEGMA and QPDMAEMA-b-POEGMA copolymer aggregates, the long chain length of OEGMA provoked a noticeable decrease in the surface charge of the P(DMAEMA-co-OEGMA) as well as of the P(QDMAEMA-co-OEGMA) copolymer aggregates. Hence, these findings suggest the efficient shielding of cationic surface charges which is crucial for effective in vitro and in vivo gene delivery.

The P(DMAEMA-co-OEGMA)-1/DNAs polyplexes were prepared in a range of N/P ratios from 0.25 to 8. The aqueous solutions of the polyplexes were transparent without the presence of precipitation. At a first glance in Figure 7, it is evident that the polyplexes formed either by the DNA of 113 bp or DNA of 2000 bp are following a similar pattern behavior, regarding the variations of the R_h_, the scattered intensity, and the zeta-potential. Specifically, in the case of the polyplexes with DNA of 113 bp, the decrease in the hydrodynamic radius (Figure 7a), in combination with the parallel decrease in the scattered intensity (Figure 7c) of the polyplexes as the N/P ratio decreases, signals the formation of polyplexes with smaller size and lower molar mass. The interaction of the copolymer aggregates with the DNA of 113 bp at the N/P ratios with the increased amount in DNA phosphate groups leads to the formation of more compact and possibly more well-defined nanostructures. On the contrary, as the N/P ratio gets higher and therefore the number of the available positive charges of the amino group increases, the R_h_ and the scattered intensity are increased. This increase demonstrates the formation of polyplexes with larger size and higher molar mass, indicating that the formation of aggregates is favored.

A similar tendency in the variations of R_h_ and scattered intensity according to the N/P ratio was also exhibited by the polyplexes with DNA 2000 bp. However, the significant differences between the polyplexes formed by the DNA of 113 bp and the DNA of 2000 bp are the resulting larger sizes and the higher scattered intensities for the polyplexes with the DNA of 2000 bp. For instance, at N/P ratio 8, the size of the polyplexes/DNA 113 bp is ca. 45 nm (close to the R_h_ of the neat copolymer aggregates), while the size of the polyplexes/DNA 2000 bp is ca. 160 nm. It should be noted that in both cases of polyplexes, a decrease in the R_h_ and the scattered intensity is observed for the N/P = 2 ratio, indicating the formation of polyplexes of different structural conformation of the components within the complexes. The interaction of the copolymer aggregates with the DNA of 2000 bp leads to the formation of nanostructures larger in size and mass, as is evident from the obtained values of the R_h_ (Figure 7b) and of the scattered intensity (Figure 7d). Consequently, the molar mass and the length of the DNA have a decisive influence on the interaction with the copolymer aggregates and on the resulting sizes of the polyplexes. Particularly, the DNA of higher molar mass can probably participate in the formation of polyplexes with a greater number of copolymer aggregates, inducing the assembly of complex nanostructures larger in size and mass.

The surface charge of the polyplexes provides important vision regarding their colloidal stability and cellular interaction. Moreover, in our case surface charge can evince the formation of polyplexes by the successful interaction of copolymer aggregates and DNAs. For the stated reasons, surface charge of the polyplexes was determined by ELS and the results obtained are presented in Figure 7e for the polyplexes with the DNA of 113 bp and Figure 7f for those with DNA of 2000 bp, at the studied N/P ratios. In most cases, polyplexes display negative charges with the apparent difference of zeta-potential absolute values according to N/P ratios. At N/P ratios below the neutralization point (N/P < 1), the polyplexes exhibit negative zeta potential of higher absolute values, due to the presence of negatively charged DNA phosphate groups in excess and which have not interacted with the positively charged amine groups of copolymer aggregates. Upon increasing the N/P ratio, the transition of the surface charge to less negative and even positive values indicates that the majority of the available positive charges of the copolymer aggregates have efficiently interacted with DNA. The polyplexes formed by the DNA of 113 bp present low negative values and almost zero compared with the surface charge of the neat copolymer aggregates of +4 mV. This phenomenon is probably assigned to the shielding effect of the non-ionic OEGMA moieties [52], which is more observable in the case of short DNA than in the case of long DNA. However, the length of OEGMA chain results in a reduction in the polyplex positive charge, a fact which is preferable to biological applications. Additionally, the negative charges in the majority of the N/P ratios and even at N/P > 1 indicate the presence of free/uncomplexed DNA molecules. This observation can be probably ascribed to the partially protonated amino DMAEMA group, which leads to weak electrostatic interactions of the copolymer aggregates with the DNAs. Furthermore, it is discerned that the polyplexes formed by the DNA of 2000 bp display strongly negative values of zeta potential at low N/P ratios, in comparison with the polyplexes prepared with the DNA of 113 bp. Specifically, at the lowest N/P ratio of 0.25, the zeta-potential value of polyplexes with the DNA of 113 bp was found to be −10 mV, while for the polyplexes of DNA 2000 bp it was found to be −27 mV. These differences in the zeta-potential values are associated with the molar mass and the conformation of the DNAs. Particularly, due to the lower molar mass of the DNA of 113 bp it is expected to have fewer negative charges in comparison with the DNA of higher molar mass which contains a greater number of negative charges. In summary, light scattering results on the formation of P(DMAEMA-co-OEGMA)-1/DNAs polyplexes revealed their efficient formation at various N/P ratios, with the length and molar mass of the DNAs and the length chain of the OEGMA segment to play a crucial role in the resulting sizes and surface charge.

Light scattering studies on the polyplexes formed by the electrostatic interaction of P(QDMAEMA-co-OEGMA)-1 copolymer aggregates and DNAs were performed on the aqueous solutions of the polyplexes at N/P ratios in the range of 0.5 to 4. During the preparation of the polyplexes, noticeable precipitation of the solutions was observed at the N/P ratios 0.6, 0.8, and 1 for the polyplexes prepared with the DNA of 113 bp and at the N/P ratios of 0.5, 0.6, 0.8, and 1 for those with the DNA of 2000 bp. However, the solutions were partially precipitated and not totally, allowing us to conduct light scattering measurements on the supernatant of the solutions at the referred N/P ratios. The electrostatic interactions caused the neutralization of the opposite charges which led to the decrease in the solubility of the polyplexes and therefore to their precipitation. However, the polyplexes formed at an excess of positive charges (N/P > 1) retained their stable state. The presence of the non-ionic OEGMA moieties prevented precipitation phenomena, providing colloidal stability to the polyplexes of N/P ratios above 1, contrary to the N/P ratios below 1, in which the support of OEGMA moieties in the colloidal stability of the polyplexes was obviously insufficient. Additionally, it is apparent that the lower content in the OEGMA (wt. 39%) segment of P(QDMAEMA-co-OEGMA)-1 copolymer affected the stability of the formed polyplexes. In contrast, the higher content of the OEGMA segment (wt. 54%) in the P(DMAEMA-co-OEGMA)-1 resulted in the formation of stable polyplexes at all N/P ratios examined.

Figure 8 depicts the obtained results by light scattering for the P(QDMAEMA-co-OEGMA)-1/DNAs polyplexes. The precipitation region is also included in Figure 8. According to Figure 8 and excluding the precipitated N/P ratios, the variations of the hydrodynamic radius and the scattered intensity are generally following a similar behavior pattern as described in the case of P(DMAEMA-co-OEGMA)-1/DNAs polyplexes. However, as the N/P ratio rises, the increase in the R_h_ is followed by a slight decrease in the scattered intensity for the polyplexes with DNA 113 bp (Figure 8a), showing the formation of polyplexes with larger size but lower molar mass. Nevertheless, the formation of more compact nanostructures as the N/P ratio decreases is observed compared with the higher N/P ratios. On the other hand, the formed polyplexes with the DNA of 2000 bp are larger in size as well as in their molar mass since by going to higher N/P ratios the rising of the R_h_ (Figure 8b) is accompanied by an increase in the scattered intensity (Figure 8c). It can also be observed, similar to the case of P(DMAEMA-co-OEGMA)-1/DNA 2000 bp polyplexes, that the sizes of the polyplexes with the DNA of 2000 bp are noticeably larger than those with the DNA of 113 bp. Due to its higher molar mass and length, DNA 2000 bp molecules can interact with a larger number of copolymer aggregates, whereas DNA of 113 bp molecules of lower molar mass can captivate a smaller number of aggregates. Bearing in mind the size of the neat copolymer aggregates (ca. 70 nm), the variations of R_h_ evidenced the formation of polyplexes.

The surface charge of P(QDMAEMA-co-OEGMA)-1/DNAs polyplexes is shown in Figure 8e,f and follows the same typical pattern as described in the case of P(DMAEMA-co-OEGMA)-1/DNAs polyplexes. However, excluding the ratios with precipitation, the transition of the surface charge of the polyplexes against N/P ratio was shifted to more positive zeta-potential values, from those of P(DMAEMA-co-OEGMA)-1/DNAs polyplexes, implying more effective interactions with the DNAs due to the strongly positive charge of the quaternary amine of the QDMAEMA segment. Moreover, it is also observed that compared with the zeta-potential value of the neat copolymer aggregates (+22 mV), the polyplexes display less positive zeta-potential absolute value which is evidence of the shielding effect of the OEGMA chains. In summary, the P(QDMAEMA-co-OEGMA)-1 copolymer aggregates effectively interacted with both DNAs and formed stable polyplexes at certain N/P ratios.

**Figure 9:**
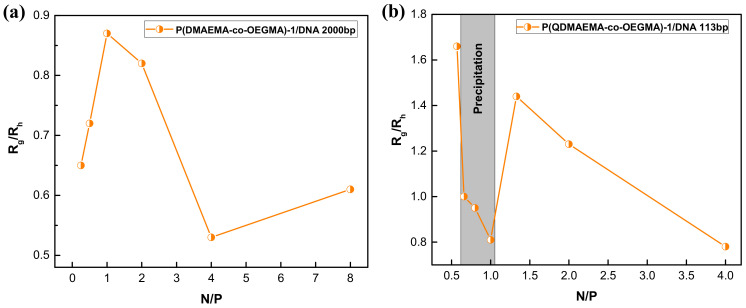
Variations of R_g_/R_h_ ratio as a function of N/P ratio for (**a**) P(QDMAEMA-co-OEGMA)-1/DNA 2000 bp and (**b**) P(QDMAEMA-co-OEGMA)-1/DNA 113 bp polyplexes.

Aiming to gain a better view on polyplexes morphology, static light scattering measurements were performed by determining the R_g_/R_h_ ratio. Indicative plots showing the variations of the R_g_/R_h_ ratio as a function of the N/P ratio for the polyplexes are given in Figure 9. In particular, Figure 9a corresponds to P(DMAEMA-co-OEGMA)-1/DNA 2000 bp polyplexes and Figure 9b to P(QDMAEMA-co-OEGMA)-1/DNA 113 bp. Concerning the polyplexes formed by P(DMAEMA-co-OEGMA)-1 copolymer aggregates and DNA of 2000 bp, the values of R_g_/R_h_ were found to obtain values from approximately 0.6 to 0.9, indicating a globular overall morphology of these polyplexes at all the studied N/P ratios. According to the overall light scattering results, DNA 2000 bp, due to its long length, tends to form more compact globular polyplexes. On the other hand, the polyplexes formed by the quaternized copolymer aggregates and the DNA of 113 bp presented R_g_/R_h_ values between 0.8 to 1.6, indicating the formation of polyplexes with more elongated structures, but also depending on the N/P ratio.

### 3.6. Influence of Ionic Strength on the Stability of the Polyplexes

A non-viral gene delivery carrier during its mission to transfer successfully and release its cargo interacts with biological fluids. The ionic strength of biological fluids affects the behavior of polyplexes regarding their colloidal and structural stability, size, mass, and surface charge. Therefore, the stability of the polyplexes under the physiological conditions of the biological fluids and in the presence of salt is essential for their effectiveness and biological performance. Hence, the consideration of these parameters is significant during the design and development of polymeric systems as efficient gene delivery vectors.

In this regard, the stability of the polyplexes under the effect of increasing solution ionic strength was examined by dynamic light scattering (DLS). To assess the tolerance of the polyplexes in the presence of salt, their size and scattered light intensity were monitored by gradually increasing the concentration of NaCl from 0.01 M (initial salt concentration of polyplexes) to 0.5 M, with the addition of NaCl 1 M solution. Moreover, due to the fact that polyplexes with a little excess of positive charges are more suitable for nucleic acid delivery and efficient intracellular uptake [1,5,6], polyplexes with N/P ratio above 1 and optimal colloidal stability for at least 1 week were selected for ionic strength investigations.

**Figure 10:**
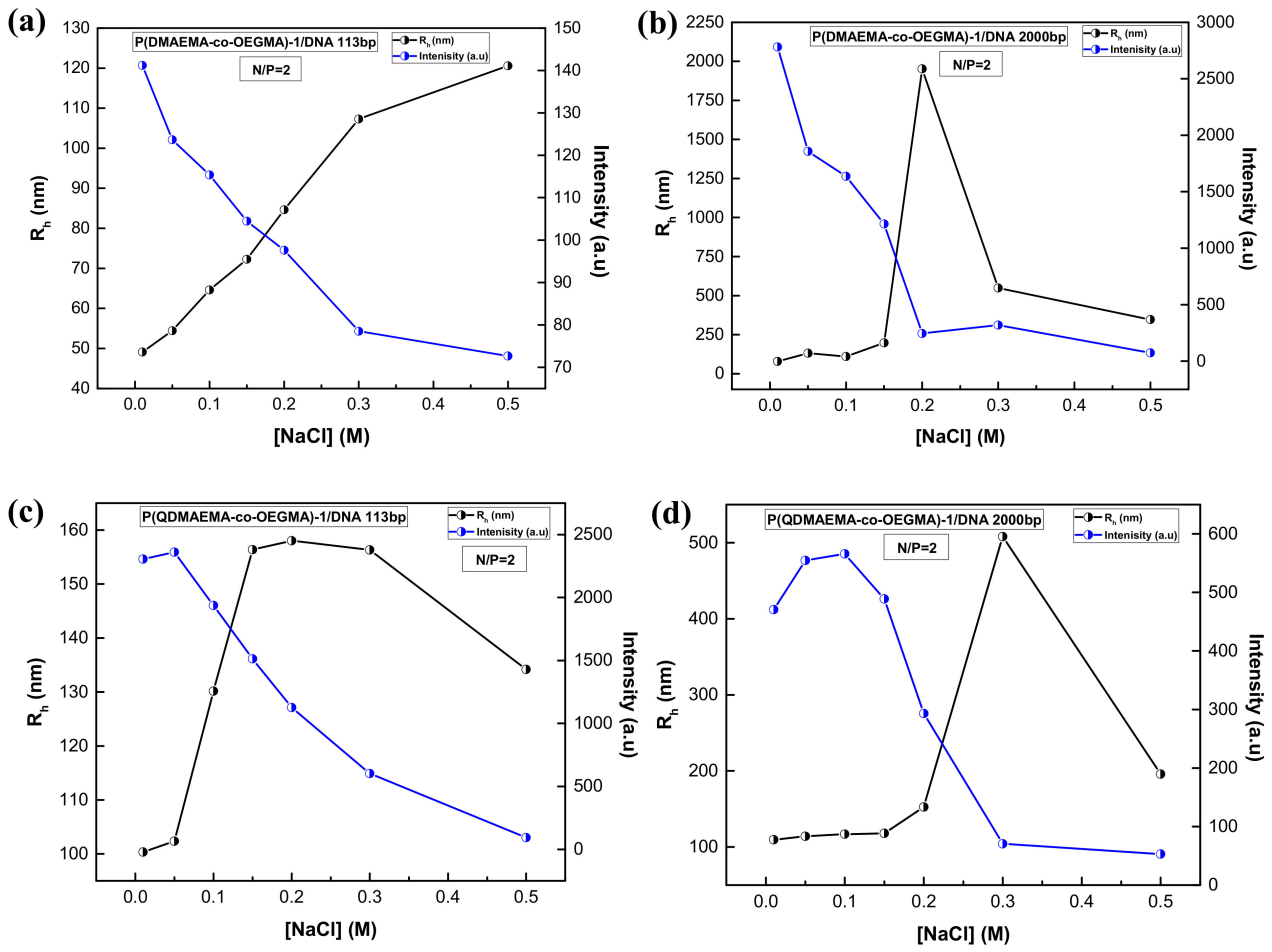
Hydrodynamic radius (R_h_) and scattered light intensity as a function of ionic strength for (**a**) P(DMAEMA-co-OEGMA)-1/DNA 113 bp, (**b**) P(DMAEMA-co-OEGMA)-1/DNA 2000 bp, (**c**) P(QDMAEMA-co-OEGMA)-1/DNA 113 bp, and (**d**) P(QDMAEMA-co-OEGMA)-1/DNA 2000 bp polyplexes at N/P = 2.

Figure 10 depicts the variations of the hydrodynamic radius (R_h_) and the scattered light intensity of P(DMAEMA-co-OEGMA)-1/DNAs and P(QDMAEMA-co-OEGMA)-1/DNAs polyplexes as a function of the ionic strength, at the ratio N/P = 2. It is observable from Figure 10 that in the presence of salt nearly all the polyplexes exhibit a gradual increase in their size (R_h_) which is followed by a parallel decrease in the scattered intensity. Particularly, P(DMAEMA-co-OEGMA)-1/DNA 113 bp polyplexes (Figure 10a) present an almost linear increase in the R_h_ and a decrease in the scattered intensity as the salt concentration rises. This increase in R_h_ signifies the growth in the size of polyplexes, while the decrease in the scattered intensity denotes the reduction in their mass. The simultaneous increase in size and decrease in mass upon increasing salt concentration implies the disintegration of the polyplexes and denotes stability issues in the presence of increased amounts of salt. The addition of salt causes charge screening effects and results in weaker electrostatic interactions between the copolymer aggregates and the DNA chains. In this way, the solubility of the polyplexes increases leading to their swelling due to enhanced insertion of water molecules within their structure.

According to Figure 10b, the P(DMAEMA-co-OEGMA)-1/DNA 2000 bp polyplexes exhibit a slight increase in R_h_ until approximately 0.15 M. However, the R_h_ increases sharply at 0.2 M and then decreases until the final salt concentration of 0.5 M. These variations along with the scattered intensity demonstrate signs of stability at low salt concentrations, but also show the swelling of the polyplexes and finally their disassociation at higher concentrations of salt.

As it can be observed in Figure 10c, the polyplexes formed by the quaternized P(QDMAEMA-co-OEGMA)-1 copolymer aggregates with the DNA of 113 bp depict similar behavior with the P(DMAEMA-co-OEGMA)-1/DNA 113 bp polyplexes. However, in this case the increase in R_h_ is more abrupt until 0.15 M, then remains constant until 0.3 M and finally declines. On the other hand, the scattered intensity is gradually decreased until the final salt concentration of 0.5 M. These changes also denote that the polyplexes lack stability upon increasing salt concentration.

The P(QDMAEMA-co-OEGMA)-1/DNA 2000 bp polyplexes in Figure 10d also exhibit similar behavior with the P(DMAEMA-co-OEGMA)-1/DNA 2000 bp. The polyplexes remain stable approximately until 0.2 M and then collapse upon the influence of increasing ionic strength. Furthermore, the polyplexes formed by DNA of 2000 bp display larger values of R_h_ as the ionic strength rises, compared with the R_h_ values of the polyplexes with the DNA of 113 bp. Upon increasing salt concentration, the electrostatic repulsions are strongly screened and the long chains of DNA 2000 bp can participate in the formation of aggregates by their complexation with a larger number of polyplexes. On the other side, the short chains of DNA 113 bp also participate in the formation of aggregates but with a smaller number of polyplexes. Thus, the aggregated polyplexes of DNA 113 bp are still smaller compared with those of DNA 2000 bp, as is evident from the R_h_ values.

Consequently, the results obtained by light scattering revealed the significance of the increase in ionic strength in the colloidal stability of the examined polyplexes as well as the impact of DNA length. The polyplexes retain their complexation ability and present colloidal stability under physiological ionic strength (equivalent to ca. 0.15 M NaCl). Moreover, the OEGMA segment probably affected the stability of the polyplexes and prevented aggregation which could result in precipitation phenomena.

## 4. Conclusions

In this study, the copolymerization of 2-(dimethylamino)ethyl methacrylate) with oligo(ethylene glycol) methyl ether methacrylate by RAFT polymerization and their further modification via quaternization of their tertiary amine group resulted in two well-defined double hydrophilic random P(DMAEMA-co-OEGMA) precursor, weakly cationic, copolymers and two P(QDMAEMA-co-OEGMA) quaternized, strongly cationic, derivatives, with desirable molecular characteristics as SEC, ^1^H-NMR, and FTIR techniques revealed.

P(DMAEMA-co-OEGMA) and their P(QDMAEMA-co-OEGMA) derivatives were investigated for their potential as nucleic acid delivery agents. In this regard, the cytotoxicity of their aggregates was evaluated in the HEK293 cell line. Among the examined copolymer aggregates, only the P(DMAEMA-co-OEGMA)-1 exhibited high cell viability in the whole range of the tested concentrations. The P(QDMAEMA-co-OEGMA)-1 copolymer aggregates presented enhanced cytotoxicity probably due to the permanent positive charge of the quaternary amine group. The encouraging biocompatibility results of P(DMAEMA-co-OEGMA)-1 led to further exploration of their cytotoxicity effect on 4T1, MDA-MB-231, MCF-7, and T47D breast cancer cell lines. The high percentage of cell viability (~90% at 150 μg/mL), which P(DMAEMA-co-OEGMA)-1 copolymer aggregates displayed in all the examined cell lines, provides perspectives for biological applications of these polymeric systems.

The ability of P(DMAEMA-co-OEGMA) and P(QDMAEMA-co-OEGMA) copolymer aggregates to electrostatically bind and condense DNA of different lengths into polyplexes was firstly confirmed by fluorescence and UV–vis spectroscopy. The findings of spectroscopy techniques, and according to the quenching and the displacement rate of ethidium bromide and also of the appearance of the UV/vis absorption peak at 225 nm (complexed DNA), revealed the stronger and effective binding affinity of the quaternized P(QDMAEMA-co-OEGMA)-1 copolymer aggregates. Moreover, P(DMAEMA-co-OEGMA)-1 copolymer aggregates interact efficiently with DNAs and form polyplexes, but the electrostatic interactions are weak due to the partially positively charged tertiary amine and furthermore due to shielding of the positive charges by the OEGMA chains.

The formation of polyplexes was also proved by light scattering techniques including DLS, ELS, and SLS. Both types of copolymer aggregates formed nanosized polyplexes in a wide range of N/P ratios. The variations of their size, scattered intensity, surface charge, and R_g_/R_h_ ratio compared with the neat copolymer aggregates demonstrate the efficient formation of colloidally stable polyplexes, at certain N/P ratios and particularly above 1 for the P(QDMAEMA-co-OEGMA)-1/DNAs polyplexes. The electrostatic interaction of both types of copolymer aggregates with the DNA of 2000 bp resulted in polyplexes with larger size and mass, compared with those with the DNA of 113 bp. Moreover, the physicochemical characteristics of polyplexes were strongly dependent on the N/P ratio. In most cases, polyplexes with smaller sizes and masses were detected at N/P ratios below 1, while upon increasing N/P ratio polyplexes with larger sizes and masses were observed, indicating the formation of more compact nanostructures at low N/P ratios. This observation was also noticed by variations of the R_g_/R_h_ ratio. Furthermore, the transition of the surface charge from negative values at N/P ratios with excess of phosphate DNA groups to positive zeta potential values upon increasing N/P also verified the efficient interaction and formation of polyplexes. Moreover, the surface charge of the polyplexes was also affected by the shielding effect of OEGMA long chains. The stability of the polyplexes under the effect of solution ionic strength was investigated by DLS. The influence of increasing ionic strength was significant in the colloidal stability of polyplexes, causing their dissociation at high salt concentration. However, polyplexes with the DNA of 2000 bp were particularly found to tolerate partially an increase in ionic strength and to retain their stability under physiological salinity.

The overall results of light scattering and also spectroscopy techniques revealed that the efficient interaction of the copolymer aggregates with DNAs and the formation of stable polyplexes are highly related to the molecular characteristics of the synthesized copolymers, i.e., molar mass, composition and architecture, the nature of the amine group, the contribution of OEGMA chain length in colloidal stability and shielding of the positive charges, the N/P ratio, and the length and molar mass of the DNA. Overall, it is observed that many factors affect the design of an effective gene delivery system. In our case, for example, P(QDMAEMA-co-OEGMA)-1 copolymer aggregates exhibited great binding affinity to DNAs, but at the same time were found to be cytotoxic. Contrarily, P(DMAEMA-co-OEGMA)-1 copolymer aggregates presented enhanced biocompatibility but also displayed weak complexation ability. Therefore, the appropriate design focusing on the optimum and delicate balance of all the affecting parameters on polyplexes performance is required in order to overcome these barriers and to improve their potential as non-viral vectors for nucleic acid delivery.

## Figures and Tables

**Table 1:** Molecular characteristics of the synthesized copolymers.

Sample	M_w_ (g·mol^−1^) (×10^4^)	M_w_/M_n_^(a)^	% wt. OEGMA	% wt. DMAEMA	% wt. QDMAEMA
P(DMAEMA-co-OEGMA)-1	1.11 ^(a)^	1.14	54 ^(b)^	46 ^(b)^	-
P(DMAEMA-co-OEGMA)-2	1.28 ^(a)^	1.15	65 ^(b)^	35 ^(b)^	-
P(QDMAEMA-co-OEGMA)-1	1.56 ^(c)^	1.14	39 ^(c)^	-	61 ^(c)^
P(QDMAEMA-co-OEGMA)-2	1.70 ^(c)^	1.15	49 ^(c)^	-	51 ^(c)^

^(a)^ Determined by SEC; ^(b)^ determined by ^1^H NMR; ^(c)^ calculated, assuming 100% quaternization.

## Data Availability

The data presented in this study are available on request from corresponding authors.
